# From structural insight to molecule: Integrative molecular simulations identify candidate pyruvate kinase activators

**DOI:** 10.1371/journal.pone.0352669

**Published:** 2026-08-12

**Authors:** Doni Dermawan, Nasser Alotaiq

**Affiliations:** 1 Department of Applied Biotechnology, Faculty of Chemistry, Warsaw University of Technology, Warsaw, Poland; 2 Health Sciences Research Center (HSRC), Imam Mohammad Ibn Saud Islamic University (IMSIU), Riyadh, Saudi Arabia; University of Sahiwal, PAKISTAN

## Abstract

Pyruvate kinase (PKLR) is a key glycolytic enzyme that regulates red blood cell energy homeostasis. Although Mitapivat is the first approved allosteric activator for pyruvate kinase deficiency (PKD), its clinical utility is limited by metabolic and pharmacokinetic challenges, underscoring the need for improved analogues. This study aimed to identify novel Mitapivat-derived scaffolds with enhanced PKLR binding, stability, and drug-like properties using an integrated computational approach. A total of 190 structurally related derivatives were screened through molecular docking, pharmacophore modeling, frontier molecular orbital (HOMO–LUMO) analysis, 200 ns molecular dynamics (MD) simulations, MM/PBSA free energy calculations, ADMET profiling, and retrosynthetic feasibility assessment. Comparative analyses were performed against Mitapivat and phenylalanine, an endogenous PKLR inhibitor. Among the screened compounds, CHEMBL3729403 and CHEMBL3729860 emerged as the most promising candidates. CHEMBL3729403 exhibited the strongest docking affinity (−9.10 kcal/mol) and superior MD stability, with the lowest average RMSD (0.419 nm) and highest hydrogen-bond occupancy (1.039). MM/PBSA calculations revealed stronger binding free energies for CHEMBL3729403 (−26.39 ± 3.21 kcal/mol) and CHEMBL3729860 (−23.05 ± 3.59 kcal/mol) than Mitapivat (−21.35 ± 3.46 kcal/mol) and phenylalanine (−3.89 ± 3.91 kcal/mol). Pharmacophore analysis demonstrated conservation of the key interaction features required for PKLR activation, while HOMO–LUMO analysis revealed comparable electronic characteristics across the lead compounds, supporting their compatibility with ligand–protein interactions. ADMET profiling predicted favorable oral absorption (81.5% and 81.3%), good intestinal permeability, acceptable drug-likeness, and the absence of predicted mutagenic, tumorigenic, reproductive, or irritant liabilities for CHEMBL3729403 and CHEMBL3729860. Retrosynthetic analysis further supported their synthetic accessibility through feasible sulfonamide-coupling routes. Overall, CHEMBL3729403 and CHEMBL3729860 were identified as promising putative PKLR activators with improved predicted binding, stability, and pharmacokinetic properties compared with Mitapivat. These findings warrant further experimental validation to confirm their therapeutic potential in PKD and related metabolic disorders.

## Introduction

Pyruvate kinase deficiency (PKD) is a rare but severe inherited red blood cell disorder caused by mutations in the pyruvate kinase (PKLR) gene, leading to impaired pyruvate kinase activity, reduced glycolytic ATP production, and compromised erythrocyte survival [1,2]. Clinically, PKD manifests with chronic nonspherocytic hemolytic anemia, splenomegaly, and iron overload, often requiring lifelong transfusion support or splenectomy [3,4]. Until recently, management of PKD was limited to supportive care, with no approved disease-modifying therapies. In this context, the development of Mitapivat (Pyrukynd®) marks a significant advance in targeted therapy for hemoglobinopathies. Mitapivat is the first-in-class, orally administered small-molecule activator of PKLR, a key glycolytic enzyme that regulates energy metabolism in red blood cells [5,6]. By enhancing PKLR activity, Mitapivat improves ATP production, stabilizes red blood cell metabolism, extends erythrocyte lifespan, and reduces transfusion dependency [7,8]. The Saudi Food and Drug Authority (SFDA) recently announced the approval of Pyrukynd for the treatment of adult patients with both alpha- and beta-thalassemia, covering transfusion-dependent and non–transfusion-dependent subtypes [9]. This decision, designated under the SFDA’s Breakthrough Medicines Program, represents a regulatory milestone for thalassemia therapy and highlights the transformative potential of PKLR activation in hereditary blood disorders. The approval was supported by robust clinical evidence. Two Phase III global trials demonstrated that Mitapivat significantly increased hemoglobin levels in non–transfusion-dependent patients and substantially reduced transfusion requirements in transfusion-dependent patients, establishing a favorable benefit–risk profile [10,11]. Despite these promising results, clinical observations also noted side effects such as headache, back pain, arthralgia, nausea, and insomnia, alongside the necessity for liver function monitoring [12,13]. Furthermore, although Mitapivat has proven efficacy, its therapeutic potential raises the question of whether rational structural modifications could yield more potent and selective PKLR activators with improved safety and pharmacokinetic profiles.

Computational drug discovery offers a powerful route to address this challenge by integrating structural biology, molecular modeling, and simulation-based methods to optimize lead compounds [14–17]. In particular, structure-guided approaches allow the identification of pharmacophoric features critical for PKLR activation, while quantum chemical calculations provide insights into the electronic properties of novel derivatives [18]. Molecular dynamics (MD) simulations, combined with binding free energy analyses, further refine the understanding of ligand–protein interactions and stability under physiological conditions [19,20]. Such integrative *in silico* approaches accelerate the identification of drug-like candidates, reducing experimental costs and facilitating rational design [21]. Moreover, natural products are being explored as metabolic modulators; for example, the flavonoid isoorientin has potent antioxidant and anti-inflammatory effects in vascular models [22], highlighting the potential of multi-functional compounds in treating metabolic and hematologic disorders. Similarly, recent studies integrating network pharmacology, molecular docking, and molecular dynamics simulations have demonstrated the utility of computational approaches for elucidating the multi-target therapeutic mechanisms of complex natural products. For instance, Huaiqihuang Granules, a traditional Chinese medicine formulation, were shown to exert potential therapeutic effects against myocarditis through multiple bioactive constituents and targets, with molecular docking and MD simulations confirming stable interactions between key compounds and disease-related proteins [23]. These findings further emphasize the growing importance of integrative computational strategies for identifying and optimizing bioactive molecules acting through diverse molecular pathways, thereby supporting the application of similar approaches in the discovery of next-generation PKLR activators.

The present study was therefore designed to explore novel and more potent pyruvate kinase activators derived from Mitapivat using an integrative molecular simulation framework. Specifically, we employed 3D structure alignment and similarity analysis, molecular docking simulations, frontier orbital analysis, pharmacophore modeling, MD simulations, and binding free energy calculations using the Molecular Mechanics/Poisson–Boltzmann Surface Area (MM/PBSA) approach. In addition, we evaluated in silico pharmacokinetic and absorption, distribution, metabolism, excretion, and toxicity (ADMET) properties to assess drug-likeness and safety. This integrative strategy provides a rational basis for advancing beyond the current standard of Mitapivat and identifying next-generation PKLR activators with enhanced therapeutic potential in thalassemia and related red blood cell disorders.

## Materials and methods

### 3D structure generation and MM2 energy minimization

The chemical derivatives of Mitapivat were retrieved from the ChEMBL database [24] (a curated repository of bioactive molecules with drug-like properties) using the SwissSimilarity platform [25]. The SMILES of Mitapivat (C1CC1CN2CCN(CC2)C(=O)C3 = CC = C(C = C3)NS(=O)(=O)C4 = CC = CC5 = C4N=CC = C5) was employed as the query input. The search was restricted to bioactive compound classes, and a combined 2D and 3D similarity-based screening approach was used. An initial pool of approximately 400 compounds was retrieved, from which 190 compounds were retained based on a similarity score ≥ 0.90 and subsequently subjected to molecular docking analysis. The rationale for using ChEMBL lies in its comprehensive, high-quality repository of experimentally validated bioactive molecules. Since ChEMBL is curated from scientific literature and databases of pharmacological assays, it ensures chemical diversity while maintaining biological relevance [26]. This makes it a robust resource for identifying potential analogs and derivatives with desirable drug-like and pharmacokinetic properties, thereby increasing the likelihood of discovering promising candidates for PKLR activation. The 3D structures of Mitapivat and its identified derivatives were subsequently generated using Chem3D Ultra software version 22 (PerkinElmer, Massachusetts, USA). To ensure geometric stability and minimize unrealistic conformations, MM2 energy minimization was applied to optimize bond lengths, bond angles, torsional strain, and steric hindrance [27,28]. This refinement step is critical for generating energetically favorable conformations suitable for further computational studies, including molecular docking and simulation analyses. Each optimized derivative was validated for structural integrity before advancing to in silico screening, where its potential as a PKLR activator was further evaluated.

### 3D structure alignment and similarity analysis

To evaluate structural consistency and conformational variation among Mitapivat and its derivatives, 3D structure alignment was performed using the superimpose feature in BIOVIA Discovery Studio version 2024 (Dassault Systèmes, Vélizy-Villacoublay, France) [29]. To further assess structural relationships, pairwise similarity analysis was carried out using the Tanimoto coefficient, which quantifies similarity based on molecular fingerprints. The resulting similarity matrix was processed and visualized as a heatmap in OriginLab Pro version 2024 (OriginLab Corporation, Northampton, MA, USA) [30]. This visualization facilitated the identification of structurally related modifications while highlighting derivatives with significant deviations from Mitapivat. To explore the relationship between molecular similarity and docking outcomes, 3D box plot analyses were performed using Molecular Operating Environment (MOE) version 2024.06 (Chemical Computing Group, Montreal, Canada) [31,32]. The plots were constructed with the X-axis representing SlogP, the Y-axis representing topological polar surface area (TPSA), and the Z-axis representing molecular weight, with normalized axes activated. The primary variable of interest was free binding energy, enabling correlation between structural modifications and docking performance. The integration of similarity heatmaps and 3D box plots provided complementary insights, allowing us to identify structure–activity relationships (SARs) and determine whether specific modifications enhanced or reduced binding affinity to the target receptor. This multi-level analysis guided the prioritization of promising Mitapivat derivatives for further computational evaluation.

### Molecular docking simulations and binding affinity analysis

Molecular docking was employed to investigate the interactions between Mitapivat, its designed derivatives, and PKLR. The objective of this step was to identify the key binding residues mediating ligand–protein interactions, characterize the types of intermolecular forces contributing to binding affinity, and compare binding orientations among different derivatives. Through this analysis, we aimed to determine which structural modifications enhanced or weakened Mitapivat’s ability to interact with PKLR. The crystal structure of PKLR was obtained from the RCSB Protein Data Bank (PDB ID: 8XFD, chain A; resolution: 2.10 Å) [33]. This structure was selected because it represents a high-resolution human PKLR crystal structure co-crystallized with Mitapivat, the clinically approved allosteric activator used as the reference compound in this study. The availability of the experimentally resolved Mitapivat-bound complex enabled accurate identification of the allosteric binding pocket, validation of the docking protocol through re-docking analysis, and direct comparison of binding modes between Mitapivat and the designed derivatives. Furthermore, the excellent structural quality and completeness of the 8XFD model make it particularly suitable for structure-based drug design and molecular simulation studies. Prior to docking, the receptor structure was refined using Swiss-PdbViewer version 4.1 (Swiss Institute of Bioinformatics, Lausanne, Switzerland) [34]. Refinement steps included removal of crystallographic water molecules, energy minimization, and correction of missing side chains, ensuring that the protein model was in an optimized state for ligand binding. PKLR allosteric binding site residues were identified using PDBSum (European Bioinformatics Institute, Cambridge, UK) [35], which provides structural annotations and ligand-binding site information. The binding pocket and active-site residues were identified based on the co-crystallized Mitapivat complex using PDBsum [36]. The following residues were defined as the PKLR allosteric binding site and used as input for docking: 36, 37, 40, 321, 363, 364, 399, 400, and 404. These residues correspond to the experimentally validated allosteric binding region of PKLR and are illustrated in S1 Fig. To maintain methodological consistency, Phenylalanine was included as a reference allosteric inhibitor of PKLR [37,38], and its structure was subjected to MM2 energy minimization under the same conditions as the Mitapivat derivatives.

Docking simulations were carried out with the High Ambiguity Driven Protein–Protein Docking (HADDOCK) platform version 2.4 (University of Utrecht, Netherlands) [39]. Although HADDOCK is primarily designed for biomolecular complexes, in this study it was applied in a restraint-driven ligand-docking mode, where experimentally derived binding-site information was explicitly incorporated to guide ligand placement. Active residues identified from the co-crystallized Mitapivat–PKLR structure (PDB ID: 8XFD), including His39, Tyr400, and surrounding pocket residues, were defined as ambiguous interaction restraints (AIRs), thereby restricting the conformational search space to a biologically relevant region and compensating for the absence of ligand-specific restraints. This approach enables biologically meaningful docking by focusing sampling within experimentally validated binding regions. To rigorously validate the docking protocol, a re-docking procedure of the co-crystallized ligand (Mitapivat) was performed, yielding a near-native binding pose with an RMSD of ~0.1 Å and preservation of key hydrogen-bond interactions (e.g., His39 and Tyr400), thereby confirming the accuracy and reliability of the HADDOCK setup for this system. Docking results were evaluated based on cluster analysis and HADDOCK scoring functions. Models were grouped into clusters, with the largest cluster population considered the most representative binding mode, as a higher population indicates reproducibility and stability of the predicted interactions. The HADDOCK score, which integrates van der Waals energy, electrostatics, desolvation energy, and buried surface area, was applied to rank docking solutions. The complex with the most favorable HADDOCK score was selected as the lead binding model. Importantly, the docking-derived binding poses were further validated through downstream molecular dynamics (MD) simulations and MM/PBSA free energy calculations, ensuring that the final interpretations were supported by dynamic stability and energetics rather than docking results alone. To complement docking analysis and refine binding energy estimations, the PRODIGY (PROtein binDIng enerGY prediction) server was used. PRODIGY predicts binding free energy (ΔG, kcal/mol) based on structural features such as intermolecular contacts, desolvation contributions, and buried surface area [40]. This provided a quantitative assessment of ligand–protein affinity, adding confidence to the docking results and enabling systematic comparison across Mitapivat derivatives.

### HOMO–LUMO analysis of mitapivat derivatives

The frontier molecular orbital properties of Mitapivat and its derivatives were investigated using density functional theory (DFT) as implemented in a custom Python workflow (homo_lumo_nature.py) [41]. All molecular structures were first subjected to full geometry optimization, followed by frontier orbital calculations at the B3LYP/6–311 + G(d,p) level of theory. This computational level was selected because the hybrid B3LYP functional has been extensively validated for predicting the electronic properties, molecular geometries, and frontier orbital energies of drug-like organic compounds, while the triple-ζ 6–311 + G(d,p) basis set provides diffuse and polarization functions that improve the description of electron delocalization, heteroatom-containing functional groups, and intermolecular interaction-related electronic effects [42–44]. Moreover, B3LYP/6–311 + G(d,p) offers a favorable balance between computational efficiency and accuracy, making it one of the most widely employed approaches for comparative HOMO–LUMO investigations in medicinal chemistry and molecular design studies [45,46]. Convergence was ensured by applying tight self-consistent field (SCF) criteria with an energy threshold of 10 ⁻ ⁶ Ha and a maximum gradient of 10 ⁻ ³ Ha/Bohr [47]. Following optimization, single-point energy calculations were performed at the same level to extract orbital eigenvalues. The script automatically identified the highest occupied molecular orbital (HOMO) and lowest unoccupied molecular orbital (LUMO). It computed the HOMO–LUMO energy gap (ΔE = E_LUMO – E_HOMO), a key descriptor of electronic excitation propensity, molecular stability, and reactivity [48]. Frontier orbital iso-surfaces were generated using standard orbital visualization tools, with an isovalue of 0.02 a.u. [49,50], to illustrate spatial delocalization patterns and assess how structural modifications influence electron distribution across the molecular scaffold. This computational setup provided a consistent framework to compare the electronic effects of different Mitapivat derivatives and rationalize their potential as novel PKLR activators.

### Pharmacophore modeling

Pharmacophore modeling was carried out to identify the essential molecular features driving the binding interactions of Mitapivat and its derivatives with PKLR. This approach provides a structural framework for understanding how different chemical groups contribute to receptor recognition and ligand affinity. Models were generated using LigandScout version 4.5 (Inte:Ligand, Vienna, Austria) [51]. The software constructs three-dimensional pharmacophores by detecting and mapping key interaction features, including hydrogen bond donors (HBDs), hydrogen bond acceptors (HBAs), hydrophobic regions, and electrostatic interaction centers. These elements represent the critical determinants of ligand–protein binding specificity and stability. By applying LigandScout’s feature recognition and alignment algorithms, we were able to compare Mitapivat with its derivatives and highlight common interaction motifs. This enabled the identification of structural modifications that preserved or enhanced key pharmacophoric features.

### Molecular dynamics (MD) simulation for structural stability and interaction analysis

The MD simulation was employed to investigate the conformational dynamics, structural stability, and interaction profiles of Mitapivat and its derivatives in complex with PKLR. The MD simulations were carried out using GROMACS version 2025.2 [52]. This approach enabled a detailed assessment of ligand–protein interactions over extended timescales, providing insights into binding stability, flexibility, and energetics beyond static docking results. Ligand parameters were generated using the General Amber Force Field (GAFF2), with atomic partial charges assigned through the AM1-BCC (Austin Model 1 with Bond Charge Corrections) method [53,54]. Topology and parameter files were prepared with acpype, integrated with AmberTools21. Protein structures were parameterized using the AMBER99sb force field, ensuring consistent treatment of bonded and non-bonded interactions between ligands and receptors. The solvated system was constructed within a dodecahedral box using the SPC water model, with a minimum distance of 1.4 nm between protein atoms and the box edges. Periodic boundary conditions (PBC) were applied in all directions to simulate bulk solvent effects. The system was neutralized with counterions, and physiological ionic strength was mimicked by adding 100 mM NaCl. Non-bonded interactions were treated with a 1.2 nm cutoff for van der Waals forces, while long-range electrostatics were calculated using the Particle Mesh Ewald (PME) method for computational accuracy [55]. Prior to production, the system was energy-minimized using the steepest descent algorithm, reducing the maximum force below 1000 kJ/mol/nm to remove steric clashes and unfavorable contacts. Equilibration was conducted in two phases: (i) NVT ensemble (100 ps), where the system was restrained, and temperature was stabilized at 310 K using the Berendsen thermostat; and (ii) NPT ensemble (100 ps), where pressure was equilibrated at 1 bar with the Berendsen barostat to achieve appropriate system density. Following equilibration, a single unrestrained 200 ns production run was performed for each protein–ligand complex. This simulation length was sufficient to capture stable binding conformations and interaction dynamics for comparative analysis across all systems. Temperature control was maintained using the V-rescale thermostat, while pressure was regulated with the Parrinello–Rahman barostat, ensuring stable simulation conditions. Trajectories were recorded at two fs intervals for subsequent analysis. Trajectory post-processing and structural interpretation focused on identifying key residues, intermolecular contacts, and conformational changes. Analyses were conducted using visualization and molecular analysis platforms, including PyMOL version 3.1.3 (Schrödinger LLC, New York, USA) [56], BIOVIA Discovery Studio 2024 (Dassault Systèmes, San Diego, USA) [29], and UCSF ChimeraX (University of California, San Francisco, USA) [57]. These tools allowed for both automated and manual evaluation of interaction dynamics and structural stability.

### Molecular mechanics/Poisson–Boltzmann surface area (MM/PBSA) calculations

The binding free energies of Mitapivat and its derivatives with PKLR were quantified using the MM/PBSA approach, applied to representative frames from the MD trajectories. This method provides a detailed assessment of ligand–protein interactions by incorporating both enthalpic contributions and solvation effects into the binding affinity evaluation [58]. To ensure robust sampling of conformational space, snapshots were extracted from the full production trajectory (0–200 ns) at every frame interval (interval = 1), as specified in the gmx_MMPBSA input file, without truncation, thereby maximizing statistical sampling across the equilibrated trajectory. For each frame, three major energetic components were calculated: (i) gas-phase molecular mechanics energy (electrostatics and van der Waals contributions), (ii) solvation free energy, comprising polar contributions from the Poisson–Boltzmann continuum model and nonpolar contributions proportional to the solvent-accessible surface area, and (iii) entropy contributions were not explicitly calculated, as quasi-harmonic (qh_entropy = 0), interaction entropy (interaction_entropy = 0), and C2 entropy (c2_entropy = 0) options were disabled in the gmx_MMPBSA configuration; thus, the reported binding free energies correspond to enthalpy-dominated estimates, which are commonly used for relative ligand ranking in comparative studies. These components yield a refined representation of the driving forces underlying ligand binding. These components yield a refined representation of the driving forces underlying ligand binding. The Single-Trajectory Protocol (STP) was employed, in which the free energies of the receptor, ligand, and complex were derived from the same simulation trajectory [59–61]. This protocol assumes minimal conformational change upon binding, thereby simplifying calculations while maintaining accuracy. Free energy estimations were performed using the gmx_MMPBSA version 1.6.4 [62], integrated within the GROMACS framework. The Poisson–Boltzmann model was applied with an internal dielectric constant of 1.0 and an external dielectric constant of 80.0, while nonpolar solvation energy was estimated using a SASA-based model with predefined surface tension parameters, as specified in the input configuration. Residue-wise energy decomposition (idecomp = 1) was performed for residues within 6 Å of the ligand to identify key contributors to binding. The overall binding free energy (ΔG_binding) was calculated according to the following equation:


$$\begin{array}{c} \Delta G\_binding\;=\;\Delta G\_complex\;-\;\Delta G\_ligand\;-\;\Delta G\_receptor \end{array}$$ (1)

where ΔG_complex is the total free energy of the solvated protein–ligand complex, ΔG_ligand corresponds to the free energy of the unbound ligand in solution, and ΔG_receptor denotes the free energy of the unbound receptor. The resulting ΔG_binding values provided a quantitative measure of binding affinity, supporting the identification of derivatives with improved interaction stability compared to Mitapivat.

### In silico pharmacokinetics and ADMET evaluation

To assess the pharmacokinetic behavior and drug-likeness of Mitapivat derivatives, a combination of computational tools was employed. SwissADME (Swiss Institute of Bioinformatics, Lausanne, Switzerland) [63] was first used to predict key physicochemical properties, including molecular weight, lipophilicity (MlogP), HBD, HBA, and TPSA. These descriptors were evaluated against Lipinski’s Rule of Five, a widely accepted guideline for estimating oral bioavailability. Compounds complying with these rules were considered more likely to possess favorable absorption and permeability characteristics, supporting their potential as orally active drug candidates. SwissADME was also applied to predict interactions with cytochrome P450 (CYP) isoenzymes, providing critical insights into metabolic stability, clearance, and the risk of drug–drug interactions. To further refine candidate selection, DataWarrior version 6.4.1 (OpenMolecules, Karlsruhe, Germany) [64] was used for predictive toxicity and drug-likeness analysis. This platform integrates molecular descriptors with predictive models to evaluate multiple toxicological endpoints, including mutagenicity, tumorigenicity, reproductive toxicity, and irritant potential. Such assessments enabled early identification of structural liabilities that could compromise drug safety and development. In addition, a more detailed pharmacokinetic and ADMET profiling was conducted using QikProp (Schrödinger, New York, USA) [65]. QikProp calculated advanced molecular descriptors relevant to absorption, distribution, metabolism, and excretion, including solvent-accessible surface area (SASA), hydrophobic SASA (FOSA), hydrophilic SASA (FISA), percentage of predicted human oral absorption, QPlogHERG (cardiotoxicity risk through hERG channel inhibition), QPPCaco (Caco-2 cell permeability), QPlogBB (blood–brain barrier penetration), QPPMDCK (MDCK cell permeability), QPlogKp (skin permeability), and QPlogKhsa (serum protein binding affinity).

### Retrosynthetic design of mitapivat derivatives

Retrosynthetic analysis of Mitapivat and its potential derivatives was performed using the ASKCOS platform (https://askcos.mit.edu/) [66]. The retrosynthesis engine was configured with a Relevance Heuristic precursor scoring system to prioritize chemically meaningful starting materials, while atom mapping was carried out using RXNMapper [67]. To ensure practical feasibility, a minimum plausibility threshold of 0.001 was applied, with regioselectivity checking and near-cycle filtering enabled to avoid unrealistic disconnections. The retrosynthetic search was limited to the top 5 candidate results per iteration, balancing computational efficiency and chemical diversity. For pathway generation, Strategy 1 was employed using the template_relevance model with the Reaxys template set, capped at a maximum of 1000 reaction templates and a cumulative probability cutoff of 0.999. This workflow enabled the systematic identification of chemically plausible Mitapivat modifications, including sulfonamide, heteroaryl, and other scaffold-decorated derivatives, which were subsequently evaluated for their potential to act as novel PKLR activators through pharmacophore-guided modeling and MD simulations.

## Results

To systematically explore the potential of Mitapivat analogues as novel PKLR activators, we performed structural alignment, molecular similarity analysis, and docking-based screening of the designed derivatives. The initial chemical space was filtered based on similarity thresholds to the parent Mitapivat structure, ensuring that key pharmacophoric elements were preserved while allowing exploration of novel substituents.

### Structure alignment and similarity analysis

A total of 190 Mitapivat derivatives were identified with a similarity score ≥ 0.90, indicating high structural conservation relative to the parent scaffold. This ensured that the derivatives retained the essential pharmacophoric elements of Mitapivat (the pyrrolidine amide core, the aryl sulfonamide moiety, and the heteroaryl substituents) while introducing chemically diverse modifications that could impact binding interactions and pharmacokinetic behavior. **Table 1** highlights representative top-scoring derivatives selected based on their docking performance and interaction similarity to native Mitapivat. Notably, derivatives such as CHEMBL4297223 and CHEMBL4299940 demonstrated near-identical similarity scores (0.999), reflecting only minor positional or tautomeric variations relative to Mitapivat. In contrast, CHEMBL3728962, CHEMBL3729403, and CHEMBL3729860 (similarity scores 0.911–0.948) displayed more pronounced chemical modifications, particularly involving sulfonyl, hydroxyl, and halogen substituents.

**Table 1:** Structural details of Mitapivat and its top-performing derivatives, including similarity score, SMILES notation, and 2D representation. This table presents only the highest-ranked derivatives based on docking performance and interaction similarity to native Mitapivat. The complete list of derivatives is provided in Supplementary Table 1.

Molecule	Similarity Score	SMILES
Mitapivat	1.000	C1CC1CN2CCN(CC2)C(=O)C3 = CC = C(C = C3)NS(=O)(=O)C4 = CC = CC5 = C4N=CC = C5
CHEMBL4297223	0.999	O = C(N1CCN(CC2CC2)CC1)C1 = CC = C(NS(=O)(=O)C2 = C3N=CC = CC3 = CC = C2)C = C1
CHEMBL4299940	0.999	O = C(N1CCN(CC2CC2)CC1)C1 = CC = C(NS(=O)(=O)C2 = C3N=CC = CC3 = CC = C2)C = C1
CHEMBL3728962	0.948	CS(=O)(=O)C1 = CC(=CC = C1)C1(O)CCN(CC1)C(=O)C1 = CC = C(NS(=O)(=O)C2 = CC = CC3 = CC = CN = C23)C = C1
CHEMBL3729403	0.922	OC1(CCN(CC1)C(=O)C1 = CC = C(NS(=O)(=O)C2 = CC = CC3 = CC = C(F)N = C23)C = C1)C1 = C(Cl)C = CC = C1
CHEMBL3729860	0.911	OC1(CCN(CC1)C(=O)C1 = CC = C(NS(=O)(=O)C2 = CC = C(F)C3 = CC = CN = C23)C = C1)C1 = C(Cl)C = CC = C1

The structural analysis of Mitapivat and its high-similarity derivatives highlighted three principal regions of divergence while maintaining the conserved pharmacophoric scaffold. These modifications were primarily localized to the sulfonyl moiety, the pyrrolidine side chain, and the aromatic substituents, each contributing distinct chemical features that may influence ligand–protein interactions. One of the most prominent differences involved modifications of the sulfonyl group. Several derivatives introduced alternative sulfonamide linkages (–SO₂NH–) or varied substitution patterns on the attached aromatic ring. For example, CHEMBL3728962 incorporated a methylsulfonyl (–SO₂CH₃) group, while CHEMBL3729403 replaced this with a fluoro-substituted sulfonyl-heteroaryl system. Such substitutions are likely to alter hydrogen-bonding capacity and redistribute electron density around the pharmacophoric region, thereby influencing both recognition and stabilization of the ligand within the binding pocket. A second notable divergence was the introduction of hydroxylated side chains. In derivatives such as CHEMBL3729403 and CHEMBL3729860, hydroxyl substituents were introduced on the pyrrolidine moiety. These modifications increase molecular polarity and solvent accessibility, potentially enhancing aqueous solubility while simultaneously enabling additional hydrogen bonding with polar residues in the protein environment. Such interactions may contribute to improved binding orientation and conformational stability compared with the native Mitapivat scaffold. Finally, halogen substitution emerged as a recurrent structural theme across several derivatives. Both CHEMBL3729403 and CHEMBL3729860 displayed chlorine and fluorine substituents on their aromatic scaffolds. Halogen atoms are well known to impart steric and electronic effects, which can modulate molecular recognition, metabolic stability, and binding affinity. Specifically, halogenation can promote hydrophobic interactions and, in some instances, halogen bonding, thereby strengthening ligand–protein complementarity. These structural modifications illustrate that while the conserved Mitapivat pharmacophore remains intact across high-similarity derivatives, peripheral tuning through sulfonyl variations, hydroxylation, and halogenation introduces chemical diversity that expands interaction potential. These differences are anticipated to modulate binding affinity, structural stability, and ADMET-related properties, warranting further evaluation through molecular docking, MD, and pharmacokinetic analyses.

**Fig 1:**
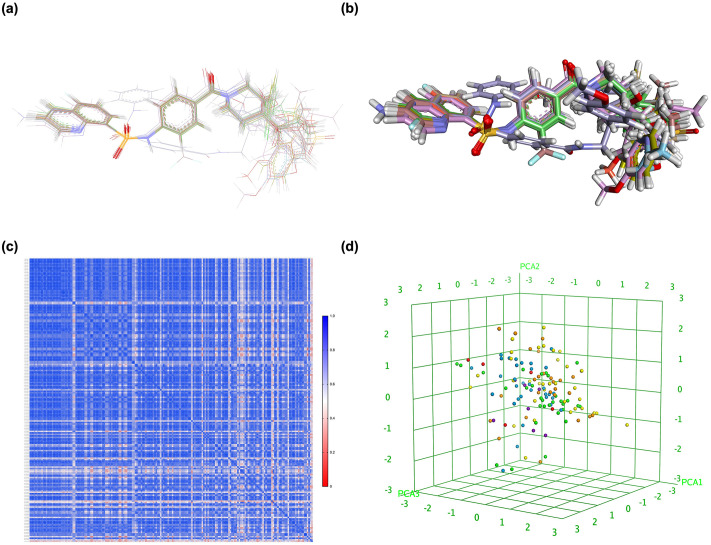
Structural alignment and similarity analysis of Mitapivat and its derivatives. **(a)** 2D alignment of Mitapivat and representative derivatives, showing conservation of the central structure with variable substituents localized to peripheral regions. **(b)** 3D conformational superposition of Mitapivat and its derivatives, highlighting consistent alignment of the aromatic and sulfonyl cores with subtle deviations in side-chain orientations. **(c)** Pairwise similarity heatmap of 190 derivatives (similarity score ≥0.90), where deep blue indicates high structural conservation and red/orange regions correspond to lower similarity clusters. **(d)** Principal component analysis (PCA) of molecular descriptors, illustrating clustering of most derivatives around the Mitapivat scaffold, with select analogues dispersed along principal components, reflecting chemical diversity introduced by substituent modifications.

**Fig 1a** illustrates the 2D-based structural alignment of Mitapivat and its derivatives, where overlapping aromatic and heteroaromatic scaffolds highlight a high degree of conservation within the central pharmacophore. This alignment emphasizes that the core structural framework, which is responsible for critical target engagement, is maintained across the derivative set. Substituent variability was predominantly localized to peripheral regions, particularly the sulfonyl moiety and pyrrolidine substituents. These modifications introduce chemical diversity while minimizing disruption of the core binding interactions. This suggests a deliberate design strategy aimed at optimizing peripheral interactions such as solubility, selectivity, or metabolic stability without compromising the essential pharmacophoric elements. **Fig 1b** shows a 3D conformational superposition, further confirming the high degree of spatial conservation across the derivative set. The aromatic core and sulfonyl-linked heterocycles were consistently aligned, while variations in hydroxyl and halogen substituents produced subtle deviations in side chain orientations. Such conformational flexibility suggests that these derivatives can occupy the binding pocket in a manner similar to Mitapivat, while still providing additional interaction opportunities through their substituent modifications.

The pairwise similarity heatmap (**Fig 1c**) quantitatively captured the structural relationships across the derivative dataset. The predominance of deep blue regions indicated that most compounds shared a high degree of similarity, consistent with the ≥ 0.90 similarity threshold applied during compound selection. This strong overall homogeneity suggests that the majority of derivatives maintain a core scaffold closely aligned with native Mitapivat. Nonetheless, localized clusters of lower similarity (red–orange traces) were evident, highlighting subsets of derivatives that diverged structurally. In particular, compounds bearing bulkier sulfonyl moieties, extended aromatic substituents, or multiple polar functional groups tended to form these lower-similarity clusters. Such deviations imply that, while still scaffold-related, these analogues introduce steric and electrostatic features that may alter binding orientation or modulate interaction profiles within the PKLR allosteric binding site. These structurally enriched outliers are therefore of particular interest, as they could represent candidates with differentiated binding behavior, potentially offering enhanced specificity, novel interaction modes, or alternative pharmacological properties. Finally, **Fig 1d** illustrates the principal component analysis (PCA) of the molecular descriptors, offering a dimensionality-reduced representation of the chemical diversity across the Mitapivat derivatives. The scatterplot revealed that the majority of compounds were tightly clustered in the PCA space, indicating strong structural conservation and conformational stability relative to the native Mitapivat scaffold. This clustering is consistent with the high Tanimoto similarity scores and superimposition analysis, confirming that most modifications preserve the pharmacophoric core of the parent molecule. In contrast, a subset of derivatives displayed noticeable dispersion along PC1 and PC3. These axes were primarily associated with variations in lipophilicity (SlogP), polarity (TPSA), molecular size (molecular weight), and electronic distribution, as identified from the 3D box plot analyses. The spread of these derivatives suggests the presence of scaffold-retaining but chemically diversified analogues, in which subtle modifications alter molecular interaction profiles without compromising overall alignment to Mitapivat. Such dispersed analogues are particularly noteworthy, as they represent structural outliers that could potentially broaden the accessible chemical space of pyruvate kinase activators.

### Molecular docking results, binding pose, and binding affinity analysis

The docking simulations provided a comprehensive assessment of the binding affinity and interaction profiles of Mitapivat and its derivatives with the PKLR allosteric binding site. As shown in **Table 2**, Mitapivat exhibited a binding energy of −8.24 kcal/mol with a HADDOCK score of −40.1, confirming its strong and specific interaction with PKLR and validating the docking protocol. The resulting binding pose closely reproduced the experimental conformation, including key interactions such as hydrogen bonding with Tyr400, with an RMSD of 0.1 ± 0.1 Å, confirming the reliability and accuracy of the docking setup. By contrast, phenylalanine, the known allosteric inhibitor of PKLR, demonstrated a weaker binding energy of −6.99 kcal/mol and a less favorable HADDOCK score (−29.7), consistent with its physiological role in negatively regulating PKLR activity. This weaker affinity not only highlights the difference between allosteric inhibition and pharmacological activation but also underscores the structural optimization achieved by Mitapivat and its analogues. Notably, several derivatives surpassed Mitapivat in docking performance. In particular, CHEMBL3729403 (−9.10 kcal/mol) and CHEMBL3728962 (−8.90 kcal/mol) showed the most favorable binding affinities, supported by HADDOCK scores of −41.8 and −45.6, respectively. These findings indicate that rational substitutions at the sulfonyl and aromatic regions enhanced stabilization within the PKLR allosteric binding site beyond that of Mitapivat itself.

**Table 2:** Comparative docking results of Mitapivat, phenylalanine (allosteric inhibitor), and top-performing derivatives with PKLR, including binding affinities and interaction energy components. The complete molecular docking results are presented in Supplementary Table 2.

Complex	HADDOCK score (a.u.)	Binding energy (kcal/mol)	Van der Waals energy	Electrostatic energy	Desolvation energy	RMSD
PKLR_Mitapivat	−40.1 ± 0.1	−8.24	−24.9 ± 0.2	−48.4 ± 2.3	−10.4 ± 0.2	0.1 ± 0.1
PKLR_Phenylalanine	−29.7 ± 0.2	−6.99	−18.6 ± 0.4	−66.8 ± 1.7	−4.5 ± 0.2	0.1 ± 0.1
PKLR_CHEMBL3729403	−41.8 ± 0.3	−9.10	−29.0 ± 0.3	−23.5 ± 1.8	−10.8 ± 0.2	0.1 ± 0.1
PKLR_CHEMBL3728962	−45.6 ± 0.3	−8.90	−31.3 ± 0.6	−41.6 ± 1.1	−10.2 ± 0.3	0.1 ± 0.1
PKLR_CHEMBL3729860	−40.7 ± 0.6	−8.85	−28.5 ± 0.5	−18.4 ± 1.4	−10.6 ± 0.2	0.1 ± 0.1
PKLR_CHEMBL4297223	−40.0 ± 0.8	−8.28	−25.1 ± 0.6	−45.5 ± 5.2	−10.4 ± 0.2	0.1 ± 0.1
PKLR_CHEMBL4299940	−40.0 ± 0.8	−8.28	−25.1 ± 0.6	−45.5 ± 5.2	−10.4 ± 0.2	0.1 ± 0.1

The energetic decomposition revealed that van der Waals forces were the primary contributors to ligand binding, with CHEMBL3728962 (−31.3 kcal/mol) and CHEMBL3729860 (−28.5 kcal/mol) exhibiting more extensive hydrophobic interactions compared to Mitapivat (−24.9 kcal/mol). Electrostatic contributions were more variable across the derivatives, with compounds such as CHEMBL3729403 and CHEMBL3729860 showing weaker electrostatic interactions than Mitapivat, suggesting that their higher binding affinities were driven primarily by steric complementarity and hydrophobic packing rather than charge-based stabilization. Desolvation energies remained consistently favorable across all complexes (approximately −10 kcal/mol), while root mean square deviation (RMSD) values of ~0.1 Å confirmed the high stability of the predicted binding poses.

**Fig 2:**
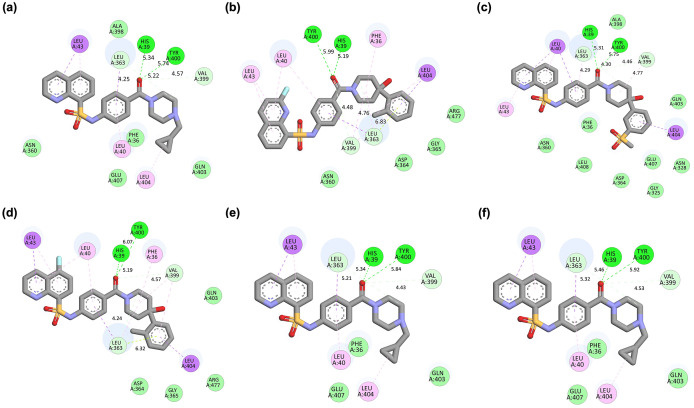
2D interaction maps of Mitapivat and its best-performing derivatives with the PKLR binding site. **(a)** PKLR_Mitapivat complex. **(b)** PKLR_CHEMBL3729403 complex. **(c)** PKLR_CHEMBL3728962 complex. **(d)** PKLR_CHEMBL3729860 complex. **(e)** PKLR_CHEMBL4297223 complex. **(f)** PKLR_CHEMBL4299940 complex. The interaction types are color-coded as follows: hydrogen bonds (bright green), carbon-hydrogen bonds (light green), van der Waals interactions (pale green), Pi-Alkyl (pink), and Pi-Sigma (purple).

The 2D interaction maps (**Fig 2**) provided detailed residue-level insights into the binding modes of Mitapivat and its derivatives within the PKLR allosteric binding site. The reference Mitapivat (**Fig 2a**) engaged in stable hydrogen bonds with His39 and Tyr400, along with carbon–hydrogen interactions involving Leu363 and Val399. These contacts are fully consistent with its reported crystallographic binding pose from the RCSB PDB database, thereby validating the re-docking procedure and confirming that the docking protocol reliably reproduced native interactions. CHEMBL3729403 (**Fig 2b**) preserved the key hydrogen bonding and carbon–hydrogen bonds observed in Mitapivat while introducing additional van der Waals interactions with Asp364, Gly365, and Arg477, which expanded the interaction density and strengthened local stabilization within the binding cleft. CHEMBL3728962 (**Fig 2c**) exhibited a broadened binding footprint through the establishment of supplementary π–π stacking interactions with aromatic residues and sulfonyl-mediated contacts. These features are likely to enhance hydrophobic complementarity and electronic stabilization. Similarly, CHEMBL3729860 (**Fig 2d**) maintained the hallmark hydrogen bonds with His39 and Tyr400 but further optimized hydrophobic packing through enhanced contacts with Leu404 and Leu43, suggesting a more favorable steric fit within the PKLR pocket. By contrast, CHEMBL4297223 (**Fig 2e**) and CHEMBL4299940 (**Fig 2f**) closely mirrored the interaction profile of Mitapivat, displaying nearly identical hydrogen-bonding and carbon–hydrogen bonding networks, which explains their comparable binding affinities (−8.28 kcal/mol each) and reinforces the robustness of the docking methodology. Importantly, only those derivatives that retained the critical hydrogen bond and carbon–hydrogen bond framework characteristic of native Mitapivat were prioritized for subsequent computational analyses, ensuring that structural modifications were evaluated within the context of preserved pharmacophoric interactions.

**Fig 3:**
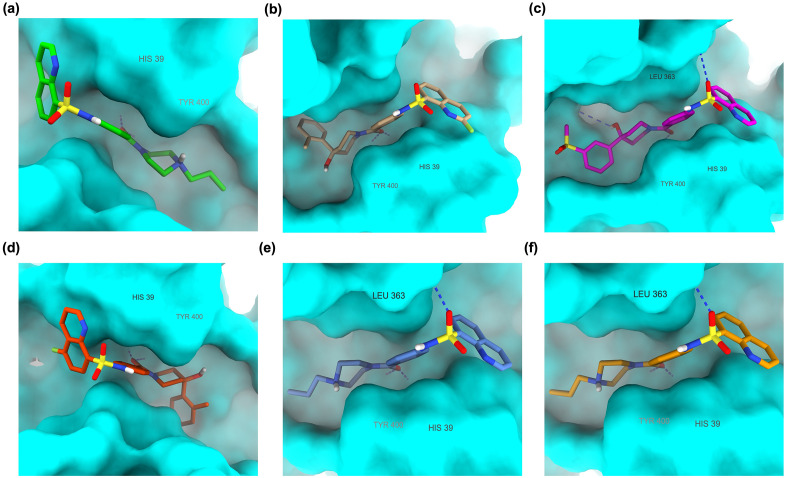
Comparative 3D binding poses of Mitapivat and its best-performing derivatives in the PKLR allosteric binding site. **(a)** PKLR_Mitapivat complex. **(b)** PKLR_CHEMBL3729403 complex. **(c)** PKLR_CHEMBL3728962 complex. **(d)** PKLR_CHEMBL3729860 complex. **(e)** PKLR_CHEMBL4297223 complex. **(f)** PKLR_CHEMBL4299940 complex.

**Fig 3** shows the comparative 3D orientations of Mitapivat and its derivatives within the PKLR allosteric binding site. The native Mitapivat (**Fig 3a**) adopts an extended conformation across the binding cleft, anchored by hydrogen bonds with His39 and Tyr400, while positioning its aromatic moiety toward the hydrophobic groove. This orientation is consistent with crystallographic data and supports its established role in stabilizing the active tetrameric conformation of PKLR. The maintenance of these anchor interactions validates the docking procedure and provides a structural reference for comparing derivative orientations. CHEMBL3729403 (**Fig 3b**) adopted a similar overall orientation to Mitapivat but displayed a slightly deeper insertion of its terminal group toward the pocket interior. This repositioning allowed it to align closely with His39 and Tyr400 while reorienting its sulfonyl group into a more favorable spatial configuration. CHEMBL3728962 (**Fig 3c**) exhibited a distinct binding posture, rotating its aromatic substituent toward Leu363, thereby broadening its footprint across the binding site. This altered orientation expanded the ligand’s coverage of the LBD, providing additional stabilization without disrupting the conserved hydrogen bond network. CHEMBL3729860 (**Fig 3d**) showed a nearly overlapping alignment with Mitapivat, retaining its planar arrangement between His39 and Tyr400 but with a subtle shift that enabled closer packing against Leu404 and Leu43. This adjustment optimized spatial complementarity within the binding cleft. In contrast, CHEMBL4297223 (**Fig 3e**) and CHEMBL4299940 (**Fig 3f**) preserved almost identical orientations to Mitapivat, with their scaffolds aligning along the same axis and engaging His39, Tyr400, and Leu363 in a manner nearly indistinguishable from the reference ligand.

The interaction profile between PKLR and its ligands revealed distinct variations in intermolecular interaction patterns across different atom pair types (**Table 3**). Mitapivat, the known allosteric activator of PKLR, displayed a total of 4,199 intermolecular contacts, with the largest contributions arising from carbon–carbon (CC, 2,083) interactions, which exclusively represent hydrophobic and van der Waals contacts and do not correspond to hydrogen bonding, and carbon–nitrogen (CN, 910) interactions, which primarily reflect polar contacts and may contribute indirectly to electrostatic stabilization but are not, by definition, hydrogen bonds unless specific geometric criteria are satisfied. Smaller contributions were observed from oxygen–oxygen (OO, 62) and oxygen–nitrogen (ON, 150) pairs, which are more directly associated with conventional hydrogen bonding interactions involving electronegative atoms. Notably, no XX-type interactions were detected in the PKLR–Mitapivat complex, indicating that Mitapivat stabilizes the protein through a combination of hydrophobic packing and polar interactions rather than extended symmetric interaction networks. In comparison, phenylalanine exhibited considerably fewer interactions, totaling only 1,910, with the majority contributed by CC (893) and CO (475) contacts. These interactions are dominated by non-polar (CC) and weak polar (CO) contacts, with CC interactions again reflecting hydrophobic/van der Waals contributions rather than hydrogen bonding. This interaction profile is therefore dominated by non-polar and limited polar interactions, with minimal contribution from atom pairs typically associated with true hydrogen bonding (i.e., involving oxygen and nitrogen atoms in appropriate geometries). The absence of CX, OX, NX, and XX interactions suggests that phenylalanine establishes a less diverse and weaker interaction network with PKLR. This reduced interaction profile is consistent with its role as a natural ligand with relatively lower binding affinity compared to allosteric activators or synthetic compounds.

**Table 3:** Molecular interaction profiles of PKLR with Mitapivat, Phenylalanine, and top-performing derivatives.

Complex	CC	CO	CN	CX	OO	OX	NO	NN	NX	XX
PKLR_Mitapivat	2083	808	910	60	62	18	150	93	15	0
PKLR_Phenylalanine	893	475	357	0	62	0	93	30	0	0
PKLR_CHEMBL3729403	2583	1032	994	107	83	26	143	62	26	2
PKLR_CHEMBL3728962	2481	1208	938	164	149	44	210	61	47	0
PKLR_CHEMBL3729860	2530	1004	974	138	82	39	141	59	40	1
PKLR_CHEMBL4297223	2104	838	935	60	62	19	151	93	16	0
PKLR_CHEMBL4299940	2104	838	935	60	62	19	151	93	16	0

### Frontier molecular orbital (HOMO–LUMO) results of Mitapivat and its derivatives

**Fig 4:**
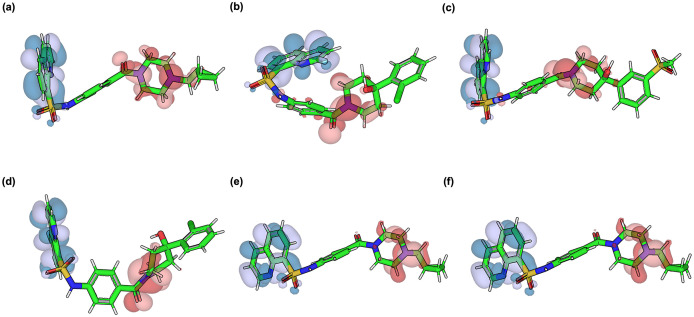
HOMO–LUMO profiles of Mitapivat and its best-performing derivatives. **(a)** Mitapivat. **(b)** CHEMBL3729403. **(c)** CHEMBL3728962. **(d)** CHEMBL3729860. **(e)** CHEMBL4297223. **(f)** CHEMBL4299940. Blue regions represent HOMO orbital localization, while red regions denote LUMO orbital distribution.

The frontier molecular orbital (FMO) analysis was performed to investigate the electronic properties of Mitapivat and its top-performing derivatives. As shown in **Fig 4**, the HOMO orbitals (blue) are predominantly localized over the aromatic scaffolds and sulfonyl groups, suggesting their role as potential electron donors during binding interactions. Conversely, the LUMO orbitals (red) are distributed across the heteroaromatic regions, indicating their ability to accept electron density from PKLR residues. This spatial distribution highlights that both the aromatic and sulfonyl regions contribute significantly to electronic complementarity with the protein’s allosteric pocket.

Mitapivat exhibited a HOMO–LUMO energy gap of 3.51 eV (**Table 4**), indicating a moderate balance between electronic stability and reactivity. The derivative CHEMBL3729403 showed a slightly larger gap of 3.59 eV, suggesting broadly comparable electronic characteristics with only marginally reduced reactivity relative to Mitapivat, alongside a reduced dipole moment (4.31 D vs. 6.37 D for Mitapivat). This reduced polarity may affect solvation and orientation within the binding pocket, while maintaining a similar electronic interaction profile. CHEMBL3728962 and CHEMBL3729860 displayed wider gaps (3.90 eV and 3.86 eV, respectively), which are more appropriately interpreted as indicating slightly higher kinetic stability and lower intrinsic chemical reactivity, rather than enhanced binding capability per se. These compounds also demonstrated redistributed HOMO densities across extended aromatic regions, which may contribute to their expanded binding footprints observed in docking studies. The relatively lower dipole moment of CHEMBL3729860 (2.93 D) suggests a more hydrophobic character that could favor tighter packing within the PKLR hydrophobic cavity. On the other hand, CHEMBL4297223 and CHEMBL4299940 showed identical electronic profiles, both with a HOMO–LUMO gap of 3.59 eV and dipole moments of 4.57 D. Their orbital distributions closely mirrored those of Mitapivat, reinforcing the observation from docking analysis that these derivatives adopt nearly indistinguishable binding modes compared to the native ligand. Overall, the relatively narrow HOMO–LUMO gap range across all compounds (3.51–3.90 eV) suggests that electronic differences are subtle and should be interpreted as supportive descriptors rather than primary determinants of binding affinity or biological activity.

**Table 4:** Frontier molecular orbital energies, HOMO–LUMO gap, and dipole moments of Mitapivat and its best-performing derivatives.

Molecule	HOMO (eV)	LUMO (eV)	Gap (eV)	Dipole (D)
Mitapivat	−5.90	−2.39	3.51	6.37
CHEMBL3729403	−6.28	−2.69	3.59	4.31
CHEMBL3728962	−6.36	−2.46	3.90	5.49
CHEMBL3729860	−6.32	−2.45	3.86	2.93
CHEMBL4297223	−5.86	−2.27	3.59	4.57
CHEMBL4299940	−5.86	−2.27	3.59	4.57

### Pharmacophore modeling results

**Fig 5:**
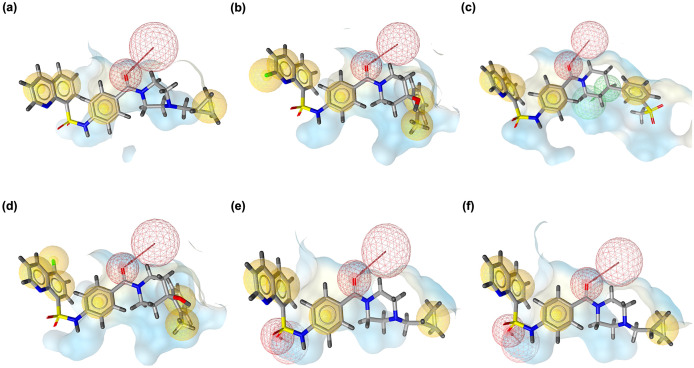
3D pharmacophore modeling. **(a)** PKLR_Mitapivat complex. **(b)** PKLR_CHEMBL3729403 complex. **(c)** PKLR_CHEMBL3728962 complex. **(d)** PKLR_CHEMBL3729860 complex. **(e)** PKLR_CHEMBL4297223 complex. **(f)** PKLR_CHEMBL4299940 complex. Yellow spheres indicate hydrophobic interactions, green arrows represent hydrogen bond donors, and red arrows signify hydrogen bond acceptors.

Pharmacophore modeling was employed to map the essential interaction features of Mitapivat and its derivatives with PKLR, thereby identifying common and distinctive binding determinants that underpin allosteric activation. As shown in **Fig 5a**, Mitapivat established a canonical pharmacophore signature characterized by two major hydrogen-bond acceptors (red spheres) directed towards polar residues of the binding cavity, complemented by multiple hydrophobic interaction hotspots (yellow spheres) anchoring the aromatic scaffold. This balanced pharmacophore arrangement confirms the well-established role of Mitapivat as a reference activator, providing both polar complementarity and strong hydrophobic packing within the PKLR allosteric site. The derivative CHEMBL3729403 (**Fig 5b**) maintained a highly similar pharmacophore map, preserving the key hydrogen-bond acceptor motif while displaying slightly expanded hydrophobic volumes towards the terminal heteroaryl region. This suggests that CHEMBL3729403 conserves the essential interaction blueprint of Mitapivat but introduces additional lipophilic stabilization. Notably, the lower dipole moment observed in electronic analyses for this compound corresponds with the reduced polarity of its pharmacophore pattern, potentially favoring hydrophobic stabilization at the expense of aqueous solubility. In contrast, CHEMBL3728962 (**Fig 5c**) exhibited a distinctive pharmacophore arrangement, with additional green features corresponding to aromatic/π-interactions. The redistribution of hydrophobic regions across the extended aromatic core indicates a broadened binding footprint, consistent with its enhanced HOMO distribution and the expanded residue contacts observed in docking analysis. This derivative, therefore, represents a promising scaffold modification capable of engaging multiple sub-pockets within the PKLR binding cleft.

CHEMBL3729860 (**Fig 5d**) revealed a compact pharmacophore distribution with strong emphasis on both hydrogen-bond acceptor features and dense hydrophobic hotspots. The reduced dipole moment of this compound, as indicated by FMO analysis, correlates with its pharmacophore profile, which shows a dominance of hydrophobic interactions over polar contacts. This hydrophobicity-driven pharmacophore suggests that 3729860 can engage PKLR more tightly through van der Waals packing, which aligns with docking observations of closer contacts against Leu404 and Leu43. Such packing interactions may compensate for the relatively fewer polar contacts, supporting its stability as an allosteric activator candidate. Similarly, CHEMBL4297223 (**Fig 5e**) and CHEMBL4299940 (**Fig 5f**) exhibited pharmacophore maps that closely mirrored Mitapivat, with conserved hydrogen-bond acceptor sites and hydrophobic clusters distributed along the central aromatic backbone. Their high degree of overlap with the Mitapivat pharmacophore highlights their role as structural mimetics rather than innovative modifications, which explains their similar electronic and docking behaviors.

### MD simulations reveal structural stability and interaction profiles of Mitapivat and its derivatives

**Fig 6:**
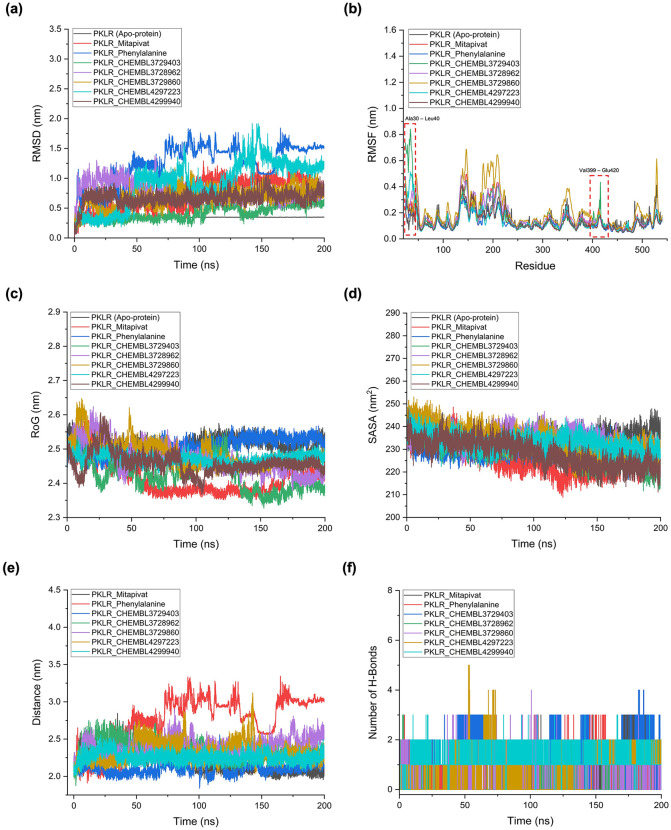
MD simulation results for PKLR in complex with Mitapivat, phenylalanine, and top-performing derivatives over 200 ns of simulation. **(a)** Root mean square deviation (RMSD), reflecting the overall conformational stability of the protein–ligand complexes. **(b)** Root mean square fluctuation (RMSF), providing residue-level insights into backbone flexibility, particularly in the active-site regions. **(c)** Radius of gyration (RoG), indicating the degree of compactness and folding stability of the protein throughout the trajectory. **(d)** Solvent accessible surface area (SASA), showing changes in surface exposure and solvation upon ligand binding. **(e)** Ligand–protein center-of-mass distance, illustrating the persistence and dynamic retention of ligands within the binding cavity. **(f)** Number of hydrogen bonds, representing the occupancy and stability of polar contacts sustaining protein–ligand interactions.

The MD simulations provided a comprehensive view of the structural and dynamic stability of PKLR in its apo form, in complex with Mitapivat, phenylalanine, and top-performing derivatives (**Table 5**, **Fig 6**). The analyses allowed a direct comparison between the known activator Mitapivat and its candidate analogs, as well as differentiation from the allosteric inhibitor phenylalanine. The RMSD analysis (**Fig 6a**) revealed that PKLR in complex with Mitapivat achieved stable equilibrium after ~30 ns with an average RMSD of 0.729 nm, reflecting moderate conformational stability. Several CHEMBL derivatives performed equally well or better. For instance, CHEMBL3729403 displayed the lowest RMSD (0.419 nm), indicating minimal deviation and high structural rigidity of the complex. Similarly, CHEMBL4299940 (0.667 nm) and CHEMBL3729860 (0.693 nm) stabilized the protein within a narrow fluctuation range, underscoring their favorable dynamic behavior. In contrast, phenylalanine exhibited significantly higher RMSD (1.244 nm), suggesting destabilization of the allosteric pocket environment, consistent with its role as an inhibitor. These findings highlight that the top-performing derivatives are capable of maintaining structural stability at levels comparable to or even superior to Mitapivat.

**Table 5:** MD simulation parameters of PKLR complexes with Mitapivat, phenylalanine, and top-performing derivatives over 200 ns of simulations, including RMSD, RMSF, RoG, SASA, ligand–protein center-of-mass distance, and hydrogen bond interactions.

Complex	Average RMSD (nm)	Average RMSF (nm)	Average RoG (nm)	Average SASA (nm^2^)	Average Distance (nm)	Number of Hydrogen Bonds Between the Ligand-Receptor
PKLR (Apo-protein)	0.347	0.145	2.515	235.361	N/A	N/A
PKLR_Mitapivat	0.729	0.156	2.414	226.448	2.200	0.708
PKLR_Phenylalanine	1.244	0.129	2.508	230.028	2.748	0.272
PKLR_CHEMBL3729403	0.419	0.167	2.425	230.302	2.107	1.039
PKLR_CHEMBL3728962	0.785	0.171	2.473	234.518	2.348	0.977
PKLR_CHEMBL3729860	0.693	0.214	2.486	233.690	2.398	0.965
PKLR_CHEMBL4297223	0.920	0.136	2.471	232.673	2.334	0.617
PKLR_CHEMBL4299940	0.667	0.135	2.458	228.195	2.257	0.752

Root mean square fluctuation (RMSF) analysis (**Fig 6b**) provided residue-level insights into flexibility changes upon ligand binding. Across most regions, Mitapivat and its derivatives showed similar fluctuation patterns, maintaining stable residues around the Ala30–Leu40 loop and the Val399–Glu420 region, both of which are critical for substrate recognition and conformational switching of PKLR. Importantly, these regions remained stabilized by hydrogen bonding in the Mitapivat and derivative complexes, whereas phenylalanine binding disrupted the stability, producing more pronounced fluctuations. Such differences emphasize that while phenylalanine interferes with allosteric pocket organization, the CHEMBL derivatives mimic Mitapivat’s stabilizing effect, reinforcing their potential role as agonists. The radius of gyration (RoG) values (**Fig 6c**) further supported these findings. Mitapivat binding reduced PKLR compactness slightly (2.414 nm), favoring a more stable fold. CHEMBL3729403 (2.425 nm) and CHEMBL4299940 (2.458 nm) produced similar compaction, whereas CHEMBL3728962 and CHEMBL3729860 yielded marginally larger RoG values (2.473–2.486 nm), which may indicate slight domain expansion. Nevertheless, the differences remained minor, and overall compactness was preserved across all active derivatives. The solvent-accessible surface area (SASA) values (**Fig 6d**) reflected the extent of protein exposure to solvent. Mitapivat produced one of the lowest SASA values (226.448 nm²), consistent with the tight packing of the ligand within the binding cavity. CHEMBL4299940 also achieved a similarly low SASA (228.195 nm²), suggesting favorable hydrophobic burial and stabilization. Meanwhile, CHEMBL3729403 and CHEMBL3728962 showed slightly higher values (230–234 nm²), yet these remained lower than phenylalanine (230.028 nm²), which is less efficient in stabilizing the protein’s hydrophobic core.

The ligand–protein center-of-mass distance plots (**Fig 6e**) indicated that Mitapivat maintained a consistent distance of ~2.2 nm throughout the simulation. CHEMBL3729403 (2.107 nm) bound even closer, demonstrating stronger anchoring within the cavity. CHEMBL4299940 (2.257 nm) and CHEMBL3728962 (2.348 nm) also remained stable, whereas phenylalanine drifted further (2.748 nm), reflecting weaker and less stable interactions. Hydrogen bond analysis (**Fig 6f**) highlighted the importance of sustained polar interactions. Mitapivat maintained an average of 0.708 hydrogen bonds, while several derivatives exhibited more persistent bonding: CHEMBL3729403 (1.039 bonds), CHEMBL3728962 (0.977 bonds), and CHEMBL3729860 (0.965 bonds). The higher hydrogen bond occupancy correlates with their superior RMSD and distance stability, confirming that these ligands form stronger and more enduring contacts within the PKLR binding site. Phenylalanine again differed, showing weaker interactions (0.272 bonds), consistent with its destabilizing effect. Among the derivatives, CHEMBL3729403 consistently outperformed Mitapivat by demonstrating the lowest RMSD, closest binding distance, and the highest number of hydrogen bonds, suggesting it is the most promising candidate for PKLR activation. CHEMBL3728962 also showed strong hydrogen bonding and acceptable structural stability, albeit with slightly higher SASA and RoG values, indicating modest structural expansion. CHEMBL4299940 emerged as another excellent candidate, combining low RMSD, favorable SASA, and strong distance stability, closely paralleling Mitapivat’s performance. Together, these derivatives not only mimic but, in certain aspects, surpass Mitapivat in maintaining PKLR structural and functional stability.

### MM/PBSA free energy analysis and per-residue decomposition

To complement the molecular docking and MD simulation findings, MM/PBSA free energy calculations were performed to quantify the binding strength of Mitapivat, phenylalanine, and the top-ranking derivatives within the PKLR allosteric binding site. The results, summarized in **Table 6**, reveal distinct energetic profiles among the tested ligands. Mitapivat displayed a binding free energy (ΔG_binding) of −21.35 ± 3.46 kcal/mol, consistent with its experimentally validated activity as an allosteric activator of PKLR. In contrast, phenylalanine, a known allosteric inhibitor, exhibited a markedly weaker interaction (ΔG_binding = −3.89 ± 3.91 kcal/mol). This weak binding energy supports its biological role as a natural but relatively modest inhibitor, insufficient to induce the strong conformational stabilization required for sustained PKLR activation. Among the derivatives, CHEMBL3729403 achieved the most favorable binding energy (−26.39 ± 3.21 kcal/mol), surpassing Mitapivat. This suggests its enhanced potential to stabilize PKLR conformations via more extensive or stronger intermolecular contacts, potentially outcompeting inhibitory ligands such as phenylalanine. Similarly, CHEMBL3729860 (ΔG_binding = −23.05 ± 3.59 kcal/mol) also outperformed Mitapivat, highlighting it as another promising scaffold. Conversely, CHEMBL3728962, CHEMBL4297223, and CHEMBL4299940 exhibited slightly weaker affinities compared to Mitapivat, though still significantly stronger than phenylalanine, reinforcing their potential to act as functional activators rather than inhibitors.

**Table 6:** MM/PBSA binding free energies (ΔG_binding) of Mitapivat, phenylalanine, and selected derivatives in complex with PKLR.

Complex	MM/PBSA Free Binding Energy ΔG_binding (kcal/mol)
PKLR_Mitapivat	−21.35 ± 3.46
PKLR_Phenylalanine	−3.89 ± 3.91
PKLR_CHEMBL3729403	−26.39 ± 3.21
PKLR_CHEMBL3728962	−20.79 ± 3.48
PKLR_CHEMBL3729860	−23.05 ± 3.59
PKLR_CHEMBL4297223	−18.49 ± 3.08
PKLR_CHEMBL4299940	−17.47 ± 3.37

**Fig 7:**
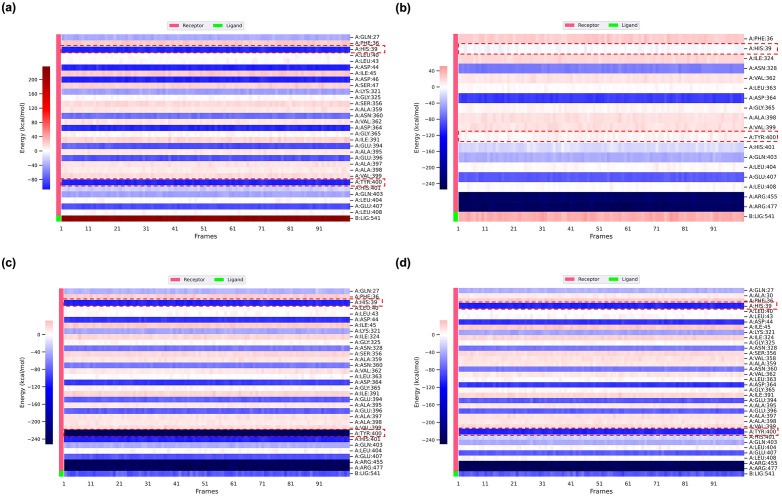
Heatmap of per-residue energy contributions (kcal/mol) in the PKLR allosteric binding site. **(a)** PKLR_Mitapivat complex. **(b)** PKLR_Phenylalanine complex. **(c)** PKLR_CHEMBL3729403 complex. **(d)** PKLR_CHEMBL3729860 complex.

Per-residue decomposition analysis, visualized in **Fig 7**, provides deeper insights into the molecular determinants of binding. Across all complexes, critical contributions were consistently observed from residues His39 and Tyr400, which align with their established roles in ligand recognition and stabilization within the PKLR allosteric binding site. Mitapivat (**Fig 7a**) engaged strongly with His39 and Tyr400, corroborating previous crystallographic findings. Phenylalanine (**Fig 7b**) exhibited significantly weaker and more diffuse interactions, particularly with His39 and Tyr400, which accounts for its reduced stabilizing effect on PKLR activity. CHEMBL3729403 (**Fig 7c**) demonstrated intensified binding contributions from His39, Tyr400, and Arg455, indicating an expanded interaction footprint that likely accounts for its superior free energy. Likewise, CHEMBL3729860 (**Fig 7d**) exhibited deep anchoring within the hydrophobic cavity, with notable stabilizing contributions from His39 and Tyr400, complemented by interactions with Glu407 and Arg455. This redistribution of interaction hotspots aligns with the observed reduction in dipole moment and enhanced hydrophobic packing suggested by FMO analysis.

### In silico pharmacokinetics and ADMET profiling of Mitapivat derivatives

To complement molecular docking, MD simulations, and MM/PBSA analyses, we further evaluated the drug-likeness and pharmacokinetic properties of the top five Mitapivat derivatives using in silico ADMET screening (**Table 7**). The compounds exhibited molecular weights between 450.55 and 565.66 g/mol, positioning them within the acceptable range of Lipinski’s “rule of five,” albeit with CHEMBL3728962 slightly exceeding the ideal upper threshold (≤ 500 g/mol). Hydrogen bond acceptors (5–7) and donors (1–2) were well-balanced across all derivatives, suggesting that these scaffolds are capable of maintaining favorable solubility and permeability. Consistent with this, the polar surface area (PSA) values ranged between 90.99–150.50 Å², with CHEMBL3728962 displaying the highest PSA, which may explain its comparatively lower predicted oral absorption. The predicted lipophilicity (cLogP) values revealed notable diversity among the derivatives. CHEMBL3729403 and CHEMBL3729860 displayed relatively high cLogP values (> 4.5), suggesting a strong hydrophobic character that may facilitate membrane penetration but potentially at the cost of solubility. In contrast, CHEMBL3728962 and CHEMBL4297223/4299940 exhibited more moderate cLogP values (2.7–2.9), indicating a balanced hydrophilic-lipophilic profile favorable for oral bioavailability. This trend is reflected in the human oral absorption scores, which were highest for CHEMBL3729403 (81.5%) and CHEMBL3729860 (81.3%), moderate for CHEMBL4297223/4299940 (78.3%), and lowest for CHEMBL3728962 (60.0%). These findings suggest that while CHEMBL3728962 retains strong binding energy against PKLR, its high PSA and reduced permeability may limit systemic exposure in vivo.

**Table 7:** In silico pharmacokinetics and ADMET properties of top-performing Mitapivat derivatives.

Parameter	CHEMBL3729403	CHEMBL3728962	CHEMBL3729860	CHEMBL4297223	CHEMBL4299940
**Molecular Weight (g/mol)**	540.01	565.66	540.01	450.55	450.55
**Hydrogen Bond Acceptors (HBA)**	6	7	6	5	5
**Hydrogen Bond Donors (HBD)**	2	2	2	1	1
**cLogP**	4.90	2.85	4.55	2.78	2.78
**Total Surface Area**	374.30	393.37	376.54	326.59	326.60
**Polar Surface Area (PSA)**	107.98	150.50	108.72	90.99	90.99
**Relative PSA**	0.210	0.277	0.215	0.218	0.219
**CYP Inhibitor**	CYP2C19, CYP2C9, CYP3A4	CYP2C19, CYP2C9, CYP3A4	CYP2C19, CYP2C9, CYP3A4	CYP2C19, CYP2C9, CYP2D6, CYP3A4	CYP2C19, CYP2C9, CYP2D6, CYP3A4
**Mutagenic**	None	None	None	None	None
**Tumorigenic**	None	None	None	None	None
**Reproductive Effective**	None	None	None	None	None
**Irritant**	None	None	None	None	None
**Shape Index**	0.541	0.538	0.540	0.594	0.594
**Molecular Flexibility**	0.465	0.493	0.466	0.470	0.470
**Molecular Complexity**	0.839	0.836	0.848	0.783	0.783
**Solvent Accessible Surface Area (SASA)**	778.635	873.227	777.041	761.733	761.733
**Hydrophobic Component of SASA (FOSA)**	149.008	193.352	149.167	294.421	294.421
**Hydrophilic Component of SASA (FISA)**	160.705	237.632	160.684	137.231	137.231
**Percent Human Oral Absorption**	81.474	60.019	81.298	78.310	78.310
**QPlogHERG**	−6.838	−7.539	−6.850	−7.304	−7.304
**QPPCaco**	296.435	55.263	296.571	123.436	123.436
**QPlogBB**	−1.312	−2.547	−1.326	−0.882	−0.882
**QPPMDCK**	240.580	21.729	218.004	57.278	57.278
**QPlogKp**	−2.515	−3.766	−2.493	−4.374	−4.374
**QPlogKhsa**	0.506	0.038	0.503	−0.038	−0.038

Evaluation of cellular permeability parameters further supports this observation. Both CHEMBL3729403 and CHEMBL3729860 achieved favorable QPPCaco (>290 nm/s) and QPPMDCK (>200 nm/s) values, indicative of efficient intestinal absorption and blood–brain barrier penetration potential. Conversely, CHEMBL3728962 displayed markedly poor values for these descriptors (QPPCaco = 55.3 nm/s, QPPMDCK = 21.7 nm/s), consistent with its high polarity. CHEMBL4297223/4299940 demonstrated moderate permeability, suggesting their oral absorption would be acceptable but not optimal compared to Mitapivat itself. Regarding distribution properties, the predicted logBB values indicated that none of the derivatives are expected to significantly cross the blood–brain barrier (−0.88 to −2.55), aligning with the therapeutic requirement for targeting red blood cell PKLR rather than central nervous system isoforms. Similarly, QPlogKp values (−2.5 to −4.4) suggest low-to-moderate skin permeability, which is not a limiting factor for oral drugs. Binding to plasma proteins, measured as QPlogKhsa, was moderate for all derivatives (0.50 for CHEMBL3729403/3729860 vs near-neutral values for CHEMBL4297223/4299940), suggesting sufficient systemic circulation without excessive sequestration.

From a metabolism perspective, all compounds were predicted to inhibit major CYP450 isoenzymes, particularly CYP2C19, CYP2C9, and CYP3A4. Interestingly, CHEMBL4297223 and CHEMBL4299940 also showed inhibition of CYP2D6, which may predispose them to drug–drug interactions in polypharmacy contexts. Nonetheless, the absence of predicted mutagenic, tumorigenic, reproductive, or irritant effects across all derivatives highlights their favorable safety profiles. A potential limitation is the negative QPlogHERG values (−6.83 to −7.53), which suggest a low but non-negligible risk of cardiotoxicity via hERG channel inhibition. Among them, CHEMBL3728962 showed the most negative score (−7.54), warranting further attention in experimental cardiotoxicity assays. Taken together, the ADMET screening highlights CHEMBL3729403 and CHEMBL3729860 as the most promising analogues, combining strong predicted oral absorption, favorable permeability, and potent binding affinity from MM/PBSA calculations.

### Retrosynthetic design of Mitapivat derivatives

**Fig 8:**
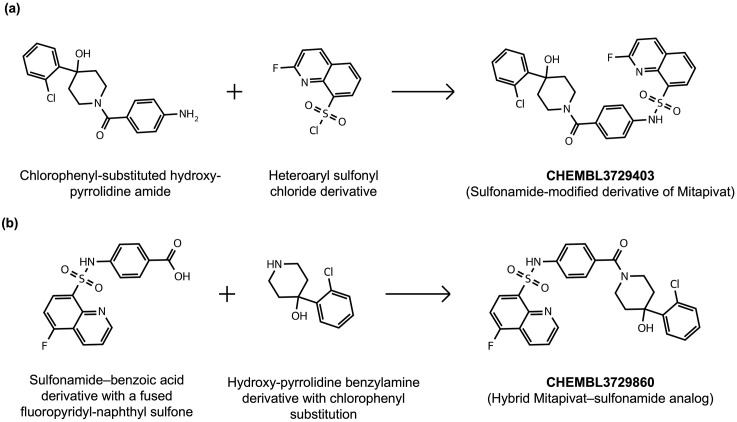
Retrosynthetic routes toward prioritized Mitapivat derivatives. **(a)** Retrosynthetic pathway leading to CHEMBL3729403, a sulfonamide-modified Mitapivat analogue, obtained via coupling of a chlorophenyl-substituted hydroxy-pyrrolidine amide with a heteroaryl sulfonyl chloride derivative. **(b)** Retrosynthetic pathway leading to CHEMBL3729860, a hybrid Mitapivat–sulfonamide analogue, synthesized through the combination of a sulfonamide–benzoic acid derivative bearing a fused fluoropyridyl-naphthyl sulfone with a hydroxy-pyrrolidine benzylamine fragment carrying a chlorophenyl substitution.

To explore rational modifications of Mitapivat toward next-generation PKLR activators, retrosynthetic analysis was performed. Among the top-ranked outputs, sulfonamide- and heteroaryl-decorated analogues emerged as particularly promising due to their favorable physicochemical and pharmacophoric alignment with the known Mitapivat scaffold. Based on their superior performance across molecular docking, MD simulations, MM/PBSA free energy calculations, and ADMET profiling, two derivatives (CHEMBL3729403 and CHEMBL3729860) were prioritized for retrosynthetic elaboration and potential synthesis. **Fig 8** illustrates two representative retrosynthetic routes leading to sulfonamide-modified Mitapivat analogues. In the first pathway (**Fig 8a**), a chlorophenyl-substituted hydroxy-pyrrolidine amide was coupled with a heteroaryl sulfonyl chloride derivative to yield CHEMBL3729403, a sulfonamide-modified derivative of Mitapivat. The introduction of the sulfonamide moiety provides enhanced hydrogen-bonding capacity and polarity, potentially improving solubility while maintaining the hydrophobic core required for PKLR binding. Furthermore, the heteroaryl substitution diversifies the aromatic pharmacophore, allowing additional π–π and polar interactions within the allosteric binding pocket identified from docking and MD simulations.

In the second retrosynthetic pathway (**Fig 8b**), a sulfonamide–benzoic acid derivative with a fused fluoropyridyl-naphthyl sulfone was combined with a hydroxy-pyrrolidine benzylamine fragment bearing a chlorophenyl group, resulting in CHEMBL3729860, a hybrid Mitapivat–sulfonamide analogue. This design introduces both extended aromatic conjugation and polar sulfonamide functionality, features predicted to strengthen binding affinity while modulating lipophilicity. Importantly, the pyrrolidine hydroxyl group is preserved, which is critical for anchoring interactions with catalytic residues in PKLR, as confirmed by the pharmacophore-guided MD analyses. The ASKCOS-guided retrosynthetic exploration not only confirmed the synthetic feasibility of these derivatives but also highlighted the chemical flexibility of Mitapivat’s scaffold. By systematically introducing sulfonamide and heteroaryl groups, we generated derivatives with improved physicochemical diversity compared to the parent drug. These structural modifications were consistent with pharmacokinetic predictions, where CHEMBL3729403 and CHEMBL3729860 demonstrated balanced lipophilicity, favorable oral absorption, and strong binding stability, underscoring their potential as lead scaffolds.

## Discussion

This study demonstrates the utility of an integrated computational pipeline combining molecular docking, pharmacophore modeling, MD simulation, MM/PBSA free energy analysis, ADMET profiling, and retrosynthetic feasibility assessment to identify next-generation derivatives of Mitapivat, a first-in-class allosteric activator of PKLR. Our findings highlight two sulfonamide-containing derivatives, CHEMBL3729403 and CHEMBL3729860, that emerged as promising scaffolds with improved binding affinities, conformational stability, and favorable pharmacokinetic properties relative to Mitapivat. Notably, these molecules also displayed significantly superior performance compared to the endogenous amino acid phenylalanine, which is known to inhibit all mammalian pyruvate kinase isozymes [68]. Pyruvate kinase catalyzes the final ATP-generating step of glycolysis, converting phosphoenolpyruvate into pyruvate [69,70]. Its regulation depends on both endogenous allosteric effectors and synthetic ligands. Phenylalanine acts as a modest negative allosteric inhibitor, binding to PKLR and preferentially stabilizing less active conformations of the enzyme [71–74]. In our study, phenylalanine exhibited only weak binding to PKLR, consistent with experimental assays showing that phenylalanine does not appreciably displace known allosteric activators at the PKLR site [75]. In contrast, Mitapivat is a potent positive allosteric activator that shifts PKLR toward the active tetrameric state, thereby restoring energy homeostasis in red blood cells from patients with pyruvate kinase deficiency [76,77]. Our computational findings confirm this dichotomy: while phenylalanine exerts only weak inhibitory effects, synthetic activators such as Mitapivat, and particularly its sulfonamide-modified derivatives, strongly stabilize the active conformation.

Despite its approval, Mitapivat is not without limitations. Clinical studies have reported variability in patient response, with some individuals showing only partial improvement in hemoglobin levels or ATP restoration [78,79]. Its long-term use has also been associated with adverse events such as headache, insomnia, and mild gastrointestinal disturbances, and emerging evidence suggests possible drug–drug interactions due to CYP450 modulation [5,80]. Furthermore, Mitapivat’s metabolic stability and solubility posed significant challenges during its development, requiring multiple rounds of medicinal chemistry optimization [81]. Importantly, resistance to Mitapivat in specific PKLR mutant variants has been reported, limiting its universal applicability across different genotypes of pyruvate kinase deficiency [77].

To ensure consistency across computational outputs, candidate prioritization was performed using a consensus-based evaluation integrating docking performance, MD-derived structural stability, MM/PBSA binding free energy, and ADMET characteristics. This multi-criteria framework enabled objective selection of lead compounds based on their overall performance rather than reliance on a single method. While initial docking results identified CHEMBL3729403 and CHEMBL3728962 as top-scoring candidates, MD simulations further indicated that CHEMBL4299940 exhibited stable binding behavior within the PKLR allosteric pocket. However, MM/PBSA free energy analysis, which provides a more rigorous estimation of binding affinity, consistently favored CHEMBL3729403 and CHEMBL3729860. Importantly, integration of ADMET properties revealed that CHEMBL3729860 possesses superior permeability and oral absorption compared to CHEMBL3728962, which exhibited limitations associated with high polarity and reduced membrane permeability. Similarly, despite its favorable MD stability, CHEMBL4299940 showed weaker binding energetics and less optimal interaction profiles. Taken together, this consensus-ranking approach explains the final selection of CHEMBL3729403 and CHEMBL3729860, as these compounds demonstrated the most balanced performance across binding affinity, structural stability, and pharmacokinetic properties. This integrative strategy reduces method-specific bias and strengthens the robustness and reproducibility of the computational conclusions.

Docking and MD simulations revealed that Mitapivat and its sulfonamide derivatives interact through a combination of hydrogen bonding, hydrophobic contacts, and π–π stacking within the PKLR allosteric pocket. CHEMBL3729403 showed persistent hydrogen bonding with key residues, including His39 and Tyr400, while maintaining hydrophobic stabilization of the binding cavity. Such interactions are consistent with crystallographic data showing that bridging the A- and B-domains enhances tetramer stability [33]. MM/PBSA analysis further highlighted that CHEMBL3729403 and CHEMBL3729860 achieved stronger binding free energy (ΔG_binding = −26.39 kcal/mol and −23.05 kcal/mol, respectively) than Mitapivat (−21.35 kcal/mol), underscoring the importance of the sulfonamide group in providing additional polarity and solvation stability while complementing hydrophobic packing. This balance of interactions is well recognized as a key determinant of effective allosteric modulation [82].

Drug development success requires not only strong target engagement but also favorable pharmacokinetic behavior and synthetic tractability. In silico ADMET profiling predicted that CHEMBL3729403 and CHEMBL3729860 possess good oral absorption, metabolic stability, and overall drug-like characteristics. However, several potential liabilities were also identified that warrant careful consideration. All evaluated compounds, including the top candidates, showed predicted inhibition of major CYP450 isoenzymes, indicating a potential risk for drug–drug interactions. Additionally, negative QPlogHERG values suggest a low but non-negligible risk of cardiotoxicity associated with hERG channel inhibition, which is an important safety concern in drug development. Furthermore, CHEMBL3728962, despite its strong docking performance, exhibited comparatively weaker permeability, likely due to its higher polarity, which may limit its bioavailability. These observations highlight the importance of balancing potency with safety and pharmacokinetic constraints during lead optimization.

Retrosynthetic analysis confirmed that both derivatives are accessible through feasible routes involving sulfonamide coupling reactions, supporting their practicality for laboratory synthesis. This synergy between retrosynthetic AI-guided design and pharmacological modeling exemplifies the growing role of computational tools in accelerating small-molecule discovery [83]. Although Mitapivat is currently approved for the treatment of pyruvate kinase deficiency (PKD), there is growing recognition that PK activators may have broader therapeutic applications. In erythrocytes, PKLR activation restores ATP levels, reduces hemolysis, and improves oxygen transport [7]. Beyond hematology, stabilization of pyruvate kinase activity has been implicated in redox balance regulation, suppression of lactate accumulation, and control of cancer metabolism through PKM2 tetramer stabilization [84,85]. The superior binding and predicted pharmacokinetic properties of CHEMBL3729403 and CHEMBL3729860 thus suggest their potential as scaffolds not only for PKD but also for metabolic disorders and oncology applications.

## Limitations and future works

Despite the promising insights derived from our computational approach, several limitations must be acknowledged. First, while molecular docking and MD simulations provide valuable predictions of ligand–protein interactions, these methods cannot fully replicate the dynamic complexity of biological systems. Our MD simulations, limited to 200 ns, successfully captured stable conformations of the ligand–PKLR complexes but may not reflect slower conformational transitions or rare events occurring on the microsecond to millisecond timescales. Additionally, the MM/PBSA binding free energy calculations, though widely used for ranking ligand affinities, inherently simplify entropic contributions and solvation effects, which may lead to deviations from experimentally measured thermodynamic values. Another limitation concerns the ADMET predictions, which were performed entirely in silico. While the computational models suggest favorable absorption, distribution, metabolism, and excretion profiles for CHEMBL3729403 and CHEMBL3729860, these predictions require validation through experimental pharmacokinetic studies in vitro and in vivo. Moreover, off-target effects, potential drug–drug interactions, and long-term safety profiles were not assessed in the present study. These parameters are particularly important for small molecules that act on metabolic enzymes, as systemic modulation of pyruvate kinase activity could alter glycolytic flux in non-target tissues. Importantly, we emphasize that all findings presented in this study are derived from computational modeling and should therefore be interpreted as predictive and hypothesis-generating rather than definitive evidence of biological activity. While the integrative pipeline improves confidence through cross-validation of multiple *in silico* methods, it does not replace experimental verification.

In terms of synthetic feasibility, our retrosynthetic analysis indicated that both derivatives can be synthesized through accessible routes. However, retrosynthetic algorithms often do not account for practical considerations such as reaction yield, stereochemical purity, cost of reagents, or scalability for industrial production. Importantly, future work should include experimental validation of the proposed compounds, including chemical synthesis under optimized conditions, determination of biological activity through enzyme-based assays (e.g., IC₅₀ or EC₅₀), and evaluation of pharmacokinetic behavior through in vivo ADME studies. In particular, direct biochemical assays measuring PKLR activation, ATP production, and enzyme kinetics will be essential to confirm the functional relevance of the predicted binding modes. Future work should involve laboratory synthesis and optimization of these derivatives, followed by biochemical assays to confirm the predicted binding affinities and functional activation of PKLR. Parallel evaluation of compound stability, solubility, and crystallinity will also be essential for successful drug development. Looking forward, future studies should include experimental validation of the computational findings through a combination of in vitro enzyme kinetics, crystallographic or cryo-EM structural studies, and red blood cell activity assays. Such experiments would confirm whether CHEMBL3729403 and CHEMBL3729860 indeed stabilize the active tetrameric conformation of PKLR and improve ATP production in patient-derived erythrocytes. Additionally, orthogonal binding assays (e.g., thermal shift assays, isothermal titration calorimetry, or surface plasmon resonance) should be employed to quantitatively validate ligand–protein interactions and binding affinities predicted in this study. Furthermore, comparative studies with existing PK activators such as Mitapivat, TEPP-46, and DASA compounds will help define the relative advantages of sulfonamide-based scaffolds. Expansion of structure–activity relationship studies may also identify additional substituents capable of further enhancing potency and selectivity. Finally, given the growing recognition that pyruvate kinase activators may have therapeutic potential beyond PKD, including other hemolytic anemias, metabolic disorders, and even cancer metabolism, future research should explore the activity of these derivatives in broader disease contexts. Long-term goals include evaluating efficacy in preclinical animal models, integrating AI-driven predictive modeling for next-generation derivative design, and ultimately advancing promising candidates into clinical testing.

## Conclusions

In conclusion, this study demonstrates the effectiveness of an integrative computational framework in identifying promising next-generation derivatives of Mitapivat, the first-in-class allosteric activator of PKLR. Our results highlight that sulfonamide-containing derivatives, particularly CHEMBL3729403 and CHEMBL3729860, exhibit stronger binding affinities, enhanced conformational stability, and more favorable pharmacokinetic profiles compared to Mitapivat, while effectively overcoming the weak inhibitory influence of the endogenous effector phenylalanine. These candidates were prioritized using a consensus-ranking strategy that integrates docking, MD simulation, MM/PBSA energetics, and ADMET properties, ensuring robust and unbiased selection across multiple computational dimensions. Importantly, the synthetic feasibility of these derivatives further supports their translational potential. Beyond identifying promising lead compounds, this work demonstrates how the integration of molecular modeling, free-energy calculations, pharmacokinetic prediction, and retrosynthetic analysis can accelerate rational drug discovery for allosteric enzyme modulators. The findings suggest that CHEMBL3729403 and CHEMBL3729860 represent promising putative PKLR activators with potential applications not only in pyruvate kinase deficiency but also in broader metabolic and hematological disorders where pyruvate kinase regulation plays a critical role. Future research should focus on experimental synthesis, *in vitro* enzyme activation assays, orthogonal binding validation studies, red blood cell functional assessments, and *in vivo* pharmacokinetic and efficacy evaluations. In addition, expanded structure–activity relationship studies, AI-guided molecular optimization, and investigations into the potential utility of PKLR activators in other disease settings may further advance the development of next-generation therapeutics. While the present findings remain computational and predictive in nature, they provide a strong foundation for future translational and experimental studies aimed at developing improved PKLR-targeted therapies.

## Supporting information

S1 Table Bioactive dataset and similarity score. (XLSX)

S2 Table Complete molecular docking results. (XLSX)

S3 Table Complete molecular interaction results. (XLSX)

S1 Fig Identification of PKLR allosteric binding site and active residues using PDBsum, based on the crystal structure of PKLR in complex with Mitapivat (PDB ID: 8XFD). (TIFF)

## Abbreviations

ADMET: Absorption, distribution, metabolism, excretion, and toxicity
AIRs: Ambiguous interaction restraints
CYP: Cytochrome P450
DFT: Density functional theory
FMO: Frontier molecular orbital
GAFF2: General amber force field
HADDOCK: High ambiguity driven protein-protein docking
HBA: Hydrogen bond acceptor
HBD: Hydrogen bond donor
HOMO: Highest occupied molecular orbital
LUMO: Lowest unoccupied molecular orbital
MD: Molecular dynamics
MM/PBSA: Molecular mechanics/Poisson-Boltzmann surface area
MOE: Molecular operating environment
NPT: Number of particles, pressure, and temperature
NVT: Number of particles, volume, and temperature
PCA: Principal component analysis
PK: Pyruvate kinase
PKD: Pyruvate kinase deficiency
PME: Particle mesh Ewald
PRODIGY: Protein binding energy prediction
RMSD: Root mean square deviation
RMSF: Root mean square fluctuation
RoG: Radius of gyration
SASA: Solvent-accessible surface area
SCF: Self-consistent field
SFDA: Saudi Food and Drug Authority
SPCE: Single point charge extended
SPR: Surface plasmon resonance
STP: Single-trajectory protocol
TPSA: Topological polar surface area.
