# Supplementary material for: From structural insight to molecule: Integrative molecular simulations identify candidate pyruvate kinase activators
Source: PLoS One. 2026 Aug 12;21(8):e0352669. doi: 10.1371/journal.pone.0352669 (PMC13466051; doi:10.1371/journal.pone.0352669)
Supplement: Supplementary file 1 [file pone.0352669.s007.pdf]

From structural insight to molecule: Integrative molecular simulations identify candidate pyruvate kinase activators		
**REVIEWER 1 COMMENTS**	**RESPONSE**	**PAGE**
**Comments 1:** The methodological concern regarding the use of HADDOCK for small-molecule docking remains insufficiently resolved. The justification provided does not adequately support its applicability in the absence of appropriate restraints, and the reliability of the reported binding modes is still questionable.	**Response 1:** We thank the reviewer for raising this important methodological concern. We acknowledge that HADDOCK is primarily developed for biomolecular docking; however, its application to small-molecule docking in the present study was carefully justified and implemented using a data-driven, restraint-guided approach based on experimentally validated binding-site residues derived from the co-crystallized Mitapivat–PKLR complex (PDB ID: 8XFD). Specifically, active residues (His39, Tyr400, and surrounding pocket residues) were defined as ambiguous interaction restraints to restrict the conformational search space to biologically relevant regions. Importantly, the reliability of this protocol was validated through successful re-docking of Mitapivat, reproducing the crystallographic binding pose with very low RMSD (~0.1 Å) and preserving key interactions, thereby demonstrating the accuracy of the docking setup even in the absence of ligand-specific restraints. Furthermore, docking predictions were not interpreted in isolation but were consistently supported by independent MD simulations, MM/PBSA free energy calculations, and interaction analyses, all of which converged on the same binding modes and key residues. Therefore, we believe that the combination of restraint-guided docking, experimental validation via re-docking, and multi-method consensus provides sufficient confidence in the reliability of the reported binding poses. The related method section was revised accordingly.	4-5
**Comments 2:** The issue related to the incorrect classification of interaction types, specifically the misinterpretation of carbon–carbon contacts as hydrogen bonds, has not been fully corrected throughout the manuscript.	**Response 2:** We thank the reviewer for highlighting this important issue. We fully acknowledge that earlier versions of the manuscript contained imprecise wording that could be interpreted as conflating carbon–carbon contacts with hydrogen bonding interactions. This has now been	14-15

	systematically corrected throughout the manuscript. In particular, we explicitly clarify that carbon–carbon (CC) interactions represent hydrophobic and van der Waals contacts only and do not correspond to hydrogen bonds, while true hydrogen bonding is restricted to interactions involving electronegative atoms such as oxygen and nitrogen (e.g., O–O, O–N, N–O pairs). We have carefully revised the text to ensure consistent and accurate classification of interaction types and removed any ambiguous phrasing. Importantly, all interpretations of binding mechanisms have been updated to reflect this corrected framework, without altering the overall conclusions of the study. These revisions ensure that the interaction analysis is now chemically rigorous and aligned with standard definitions in molecular recognition studies.	
**Comments 3:** The inconsistency in HOMO–LUMO gap interpretation persists, and the corresponding statements require careful revision to align with the reported data.	**Response 3:** We thank the reviewer for pointing out the inconsistency in the interpretation of HOMO–LUMO gaps. We have now carefully revised the manuscript to ensure that all statements are fully consistent with the reported data and standard quantum chemical interpretation. Specifically, we avoid overgeneralization by clarifying that variations in HOMO–LUMO gap within the narrow range observed (3.51–3.90 eV) indicate only modest differences in electronic stability and reactivity, rather than definitive superiority of any compound. Larger gaps are now described as reflecting slightly increased kinetic stability and lower chemical reactivity, while comparable gaps are interpreted as similar electronic behavior, without implying enhanced biological activity. Importantly, we explicitly state that FMO results are supportive and not determinative, and must be interpreted alongside docking, MD, and MM/PBSA analyses.	1, 16
**Comments 4:** The manuscript still lacks appropriate literature support for certain mechanistic claims, including allosteric inhibition by phenylalanine.	**Response 4:** We appreciate the concern. We have revised the paragraph to cite primary literature for phenylalanine's effect on PK. In particular, phenylalanine	26

	is known to inhibit all major PK isoforms (Uyeda, 2013), while more recent experimental evidence (Nilsson et al., 2025) demonstrates that phenylalanine does not significantly interfere with binding of allosteric activators at the PKLR site. Thus, in contrast to potent activators such as Mitapivat, phenylalanine's effect on PKLR is relatively modest and context-dependent. Our computational results, which show weak binding of phenylalanine compared to Mitapivat and its derivatives, are fully consistent with these experimentally reported observations. The discussion section was revised accordingly.	
**Comments 5:** Importantly, the study continues to rely entirely on computational findings without sufficient discussion of limitations or experimental validation, which weakens the strength of the conclusions.	**Response 5:** We appreciate this important comment and agree that relying solely on computational evidence can limit the strength of the conclusions if not properly contextualized. In response, we have substantially revised the Limitations and Future Works section to more explicitly acknowledge the constraints of docking, MD simulations, MM/PBSA, and in silico ADMET predictions, and to clearly position our findings as hypothesis-generating rather than confirmatory. We now explicitly outline a stepwise experimental validation framework, including compound synthesis, in vitro enzymatic assays (EC_{50}/activation studies), structural validation (X-ray/cryo-EM), red blood cell functional assays, and in vivo pharmacokinetics, to bridge computational predictions with biological evidence. These additions clarify that our study provides a rational prioritization strategy for candidate PKLR activators, while emphasizing that definitive validation requires experimental investigation.	28-29
