# Supplementary material for: From structural insight to molecule: Integrative molecular simulations identify candidate pyruvate kinase activators
Source: PLoS One. 2026 Aug 12;21(8):e0352669. doi: 10.1371/journal.pone.0352669 (PMC13466051; doi:10.1371/journal.pone.0352669)
Supplement: Supplementary file 3 [file pone.0352669.s009.pdf]

From structural insight to molecule: Integrative molecular simulations identify candidate pyruvate kinase activators		
REVIEWER 3 COMMENTS	RESPONSE	PAGE
**Comments 1:** The abstract should be improved according to the results.	**Response 1:** Thank you for this valuable suggestion. We have revised the Abstract to better reflect the main findings of the study. Specifically, we emphasized the identification of CHEMBL3729403 and CHEMBL3729860 as the top-ranked Mitapivat derivatives based on a consensus evaluation integrating molecular docking, molecular dynamics simulations, MM/PBSA binding free energy calculations, and ADMET profiling. Key quantitative results have been incorporated, while supportive analyses such as pharmacophore modeling, HOMO–LUMO calculations, and retrosynthetic assessment are presented more concisely. The revised Abstract now provides a clearer and more results-oriented summary of the study.	1
**Comments 2:** The introduction should be improved by adding recent references. These references should be added to improve it. https://doi.org/10.3390/molecules24091714 https://doi.org/10.1016/j.molstruc.2019.02.071 https://doi.org/10.1007/s10753-025-02289-2 https://doi.org/10.2174/0113816128393399251021101651 https://doi.org/10.1080/13813455.2026.2628187 https://doi.org/10.3390/antiox15010109	**Response 2:** We have substantially revised the Introduction to incorporate mentioned studies and satisfy the request for additional citations. In particular, we have added references that illustrate the effectiveness of computational drug discovery methods and highlight emerging insights into metabolic regulation. For example, we note that “various computational models have been exploited to infer drug–target interactions” and accelerate lead optimization in drug discovery.	2-3
**Comments 3:** Justify the usage of the functional and basis set (B3LYP level with the 6-311+G (d, p)) used in the calculations.	**Response 3:** We thank the reviewer for this valuable comment. The manuscript has been revised to provide a clearer justification for the selection of the DFT functional and basis set used in the HOMO–LUMO calculations. Specifically, we explain that the B3LYP functional combined with the 6-311+G(d,p) basis set represents a well-established and extensively validated level of theory for calculating frontier molecular orbitals,	5

	electronic descriptors, and charge distributions of drug-like organic molecules. The inclusion of diffuse functions (+) improves the description of electron-rich heteroatoms and delocalized π-systems, while polarization functions (d,p) enhance the accuracy of molecular geometries and orbital distributions. Furthermore, this level of theory provides a favorable balance between computational cost and predictive accuracy, making it particularly suitable for comparative electronic structure analyses of medium-sized pharmaceutical compounds such as Mitapivat and its derivatives. The corresponding explanation has been incorporated into the Methods section.	
**Comments 4:** The quality of some figures should be improved.	**Response 4:** We thank the reviewer for this helpful comment. To ensure optimal image quality for publication, all figures have been prepared and uploaded separately as high-resolution TIFF (.tiff) files through the submission portal in accordance with the journal's figure requirements. The lower visual quality observed in the manuscript file is primarily due to image compression during document preparation and PDF generation. The editorial office and production team will have access to the original high-resolution figure files for peer review and final publication. We have also carefully checked all figure labels, legends, and graphical elements to ensure clarity and readability.	All pages
**Comments 5:** What is the motivation behind the selection of the protein PDB: 8XFD?	**Response 5:** Thank you for this valuable comment. We have clarified the rationale for selecting PDB ID: 8XFD in the revised manuscript. This structure was chosen because it represents a high-resolution (2.10 Å) crystal structure of human PKLR co-crystallized with Mitapivat, the reference allosteric activator investigated in this study. The presence of the experimentally resolved Mitapivat-bound conformation allowed us to accurately define the allosteric binding pocket, validate the docking protocol	4

	through re-docking experiments, and directly compare the binding behavior of newly designed derivatives against the clinically approved ligand. Furthermore, the excellent structural quality and completeness of the 8XFD model make it particularly suitable for molecular docking, molecular dynamics simulations, and subsequent binding free energy analyses	
**Comments 6:** Finally, the conclusion section should be expanded to provide a more comprehensive summary. It should include the authors' perspectives on future research directions in this field.	**Response 6:** Thank you for this valuable suggestion. We agree that the original conclusion was relatively concise and did not sufficiently emphasize the broader implications of our findings or future research directions. Accordingly, we have expanded the Conclusion section to provide a more comprehensive summary of the study, highlight the significance of the identified lead compounds, acknowledge the predictive nature of the computational results, and outline future research priorities, including experimental validation, structure–activity relationship optimization, AI-assisted lead design, and evaluation in broader therapeutic applications beyond pyruvate kinase deficiency.	25
