# Supplementary material for: From structural insight to molecule: Integrative molecular simulations identify candidate pyruvate kinase activators
Source: PLoS One. 2026 Aug 12;21(8):e0352669. doi: 10.1371/journal.pone.0352669 (PMC13466051; doi:10.1371/journal.pone.0352669)
Supplement: Supplementary file 4 [file pone.0352669.s006.docx]

| **From structural insight to molecule: Integrative molecular simulations identify candidate pyruvate kinase activators** | | |
| --- | --- | --- |
| **EDITOR COMMENTS** | **RESPONSE** | **PAGE** |
| **Comments 1:** Please ensure that your manuscript meets PLOS ONE's style requirements, including those for file naming. The PLOS ONE style templates can be found at  <https://journals.plos.org/plosone/s/file?id=wjVg/PLOSOne_formatting_sample_main_body.pdf> and  <https://journals.plos.org/plosone/s/file?id=ba62/PLOSOne_formatting_sample_title_authors_affiliations.pdf> | **Response 1:** Thank you for your guidance. We have carefully revised the manuscript to ensure full compliance with PLOS ONE’s style requirements. Specifically, we have adjusted the formatting of the main text, title page, author names, and affiliations in accordance with the official PLOS ONE templates provided. In addition, we have reviewed and updated file naming conventions, figure and table formatting, section organization, and overall manuscript structure to align with PLOS ONE guidelines. | All pages |
| **Comments 2:** Please note that PLOS One has specific guidelines on code sharing for submissions in which author-generated code underpins the findings in the manuscript. In these cases, we expect all author-generated code to be made available without restrictions upon publication of the work. Please review our guidelines at <https://journals.plos.org/plosone/s/materials-and-software-sharing#loc-sharing-code> and ensure that your code is shared in a way that follows best practice and facilitates reproducibility and reuse. | **Response 2:** In response, we have ensured that all custom scripts used in this study (including the DFT-based HOMO–LUMO analysis workflow) are made publicly accessible without restriction. The code has been deposited in a publicly available repository (GitHub), and the corresponding link has been included in the revised manuscript under the Data Availability section. | 29 |
| **Comments 3:** Thank you for stating the following financial disclosure: “This work was supported and funded by the Deanship of Scientific Research at Imam Mohammad Ibn Saud Islamic University (IMSIU) (grant number IMSIU-DDRSP2501).”  Please state what role the funders took in the study. If the funders had no role, please state: "The funders had no role in study design, data collection and analysis, decision to publish, or preparation of the manuscript." If this statement is not correct you must amend it as needed. Please include this amended Role of Funder statement in your cover letter; we will change the online submission form on your behalf. | **Response 3:** Thank you for your comment. We would like to inform you that the grant number has been corrected to IMSIU-DDRSP2601 (due to the year of publication - 2026) in the revised manuscript. In addition, we have included the required statement regarding the role of the funder. The revised Financial Disclosure section now reads:  “This work was supported and funded by the Deanship of Scientific Research at Imam Mohammad Ibn Saud Islamic University (IMSIU) (grant number IMSIU-DDRSP2601). The funders had no role in study design, data collection and analysis, decision to publish, or preparation of the manuscript.” | 29 |
| **Comments 4:** We note you have included a table to which you do not refer in the text of your manuscript. Please ensure that you refer to Table 3 in your text; if accepted, production will need this reference to link the reader to the Table. | **Response 4:** Thank you for pointing this out. We have carefully revised the manuscript to ensure that Table 3 is now appropriately cited and discussed in the main text. The relevant section has been updated to clearly refer to Table 3, allowing proper linkage between the text and the presented data. | 14 |
| **Comments 5:** If the reviewer comments include a recommendation to cite specific previously published works, please review and evaluate these publications to determine whether they are relevant and should be cited. There is no requirement to cite these works unless the editor has indicated otherwise. | **Response 5:** Thank you for your guidance. We have carefully reviewed the reviewer comments and confirm that no specific references were suggested for inclusion. Therefore, no additional citations were required or added to the manuscript in this regard. | N/A |
| **REVIEWER 1 COMMENTS** | **RESPONSE** | **PAGE** |
| **Comments 1:** The authors used HADDOCK to dock small molecules into PKLR. while it is designed primarily for protein–protein or protein–nucleic acid docking and requires ambiguous interaction restraints (AIRs), which are derived from experimental data. the authors did not provide any AIRs. The use of HADDOCK in this context raises concerns about the accuracy and reliability of the reported binding modes and relative affinities as well as conclusions drawn for the top candidates. Redock using a standard small molecule docking tool. | **Response 1:** We thank the reviewer for this important comment regarding the use of HADDOCK for small-molecule docking.  First, we would like to clarify that blind docking was not performed in this study. Instead, docking simulations were conducted using experimentally validated active-site information derived from the crystal structure of PKLR in complex with Mitapivat (PDB ID: 8XFD). The binding site and key residues were identified using PDBsum, ensuring that docking was restricted to a biologically relevant allosteric pocket.  The following active-site residues were explicitly defined and used as input restraints in HADDOCK:  36, 37, 40, 321, 363, 364, 399, 400, and 404. These residues have now been clearly added to the revised Materials and Methods section and are also illustrated in Supplementary Figure S1.  Furthermore, we employed HADDOCK in ligand-docking mode, which is specifically adapted for protein–small molecule interactions. Although HADDOCK is widely known for protein–protein docking, it has been successfully applied to ligand docking when structural information is available, as in our case.  To validate the docking protocol, we performed re-docking of the co-crystallized ligand (Mitapivat). The results demonstrated high consistency with the experimental binding pose, including preservation of key interactions such as hydrogen bonding with Tyr400, and yielded an RMSD of 0.1 ± 0.1 Å, indicating excellent agreement with the crystallographic structure.  This successful re-docking validation confirms the reliability of our docking setup. Therefore, we believe that the use of additional docking tools is not strictly necessary, as the current protocol already demonstrates high accuracy, reproducibility, and agreement with experimental data.  We have clarified these points in the revised manuscript to improve methodological transparency and rigor. | 4, 11 |
| **Comments 2:** The hydrogen bond definition and count are unclear. the manuscript repeatedly refers to carbon-carbon (CC) contacts as "hydrogen bond interactions". CC contacts are non-polar, hydrophobic, or van der Waals interactions, not hydrogen bonds. | **Response 2:** We thank the reviewer for this important clarification. We fully agree that carbon–carbon (CC) contacts do not represent hydrogen bonds, but rather correspond to non-polar, hydrophobic, or van der Waals interactions.  In the original manuscript, the term “hydrogen bond interactions” was inadvertently used as a general descriptor for all intermolecular contacts categorized by atom pair types. We acknowledge that this terminology was inaccurate and may lead to confusion.  To address this, we have carefully revised the manuscript to:   1. Replace the term “hydrogen bond interactions” with “intermolecular interactions” where appropriate. 2. Clearly distinguish between true hydrogen bonds (e.g., involving O and N atoms) and non-polar contacts (e.g., CC interactions). 3. Update the corresponding text in the Results section to reflect the correct chemical interpretation. | 14-15 |
| **Comments 3:** The abstract claims that CHEMBL3729403 "exhibited the smallest HOMO-LUMO gap". This is incorrect based on the data in Table 4. Mitapivat has a gap of 3.51 eV, while CHEMBL3729403 has a larger gap of 3.59 eV. This error in the abstract must be corrected. | **Response 3:** We thank the reviewer for carefully identifying this inconsistency. We acknowledge that the statement in the original abstract incorrectly described CHEMBL3729403 as having the smallest HOMO–LUMO gap. Based on the data presented in Table 4, Mitapivat exhibits the smallest gap (3.51 eV), while CHEMBL3729403 shows a slightly larger value (3.59 eV). We have corrected the abstract accordingly to accurately reflect the results and avoid misinterpretation. The revised text now appropriately describes the electronic properties of CHEMBL3729403 without overstating its HOMO–LUMO gap relative to Mitapivat. | 1 |
| **Comments 4:** Provide a reference for allosteric inhibition of PKLR-type by phenylalanine | **Response 4:** We have added appropriate references that clearly demonstrate the role of phenylalanine as a negative allosteric regulator of pyruvate kinase. These references are now incorporated into the revised manuscript to strengthen the scientific basis of our methodology. | 4 |
| **Comments 5:** It's better to support the results of computational studies with experimental data as (IC50, synthetic condition and in vivo ADME profile) | **Response 5:** We thank the reviewer for this important suggestion. We fully agree that experimental validation, including IC₅₀ determination, synthetic procedures, and in vivo ADME profiling, is essential to confirm the pharmacological potential of the proposed compounds.  However, the primary objective of the present study was to perform a comprehensive multiscale in silico investigation, integrating molecular docking, pharmacophore modeling, MD simulations, MM/PBSA free energy calculations, ADMET prediction, and retrosynthetic analysis to prioritize promising Mitapivat derivatives. As such, experimental validation was beyond the scope of this work.  To address this limitation, we have explicitly incorporated the reviewer’s suggestion into the “Limitations and future works” section, where we now emphasize the necessity of future experimental studies, including biochemical assays (e.g., IC₅₀), synthetic validation, and in vivo pharmacokinetic evaluation. | 27-28 |
| **REVIWER 2 COMMENTS** | **RESPONSE** | **PAGE** |
| **Comments 1:** The title and parts of the abstract/conclusion imply that the study has “revealed novel pyruvate kinase activators,” but the work is entirely computational. The manuscript itself states that biochemical, cellular, and in vivo validation are still needed to confirm efficacy and safety. The claims should therefore be softened throughout to “candidate,” “putative,” or “predicted” PKLR activators unless experimental validation is added. | **Response 1:** We thank the reviewer for this important and valid comment. We agree that, as this study is entirely computational, the terminology should be carefully framed to avoid overstating the findings. Accordingly, we have revised the title, abstract, and conclusions to replace definitive terms such as “activators” with more appropriate language including “candidate,” “putative,” and “predicted” PKLR activators. These changes ensure that the claims are fully aligned with the in silico nature of the work while maintaining scientific accuracy and rigor. | 1, 28 |
| **Comments 2:** The overall workflow is described, but key operational details are still missing or too general. The manuscript should clarify: how the 190 compounds were reduced to the final shortlisted set; the exact docking restraints and cluster-selection logic in HADDOCK; whether MD simulations were run once or replicated; how MM/PBSA frames were selected; how entropy was actually estimated; and whether the custom homo_lumo_nature.py script and relevant inputs will be shared. These details are important for a study whose conclusions depend heavily on computational ranking. | **Response 2:** We thank the reviewer for this constructive comment, highlighting the need for greater methodological transparency. In response, we have clarified several key operational aspects of the workflow to improve reproducibility and rigor:   1. Compound selection: We now explicitly state that the initial pool of ~400 compounds retrieved from SwissSimilarity was filtered using a similarity threshold (≥ 0.90), resulting in 190 compounds subjected to docking. 2. Docking restraints and selection: The active-site residues derived from the co-crystallized structure (PDB ID: 8XFD) via PDBsum were used as restraints in HADDOCK ligand-docking mode. Cluster selection was based on both cluster size (population) and HADDOCK score, with the most populated and energetically favorable cluster selected. 3. MD simulations: We clarify that a single 200 ns production run per system was performed following proper equilibration. 4. MM/PBSA frame selection: Frames were extracted from the full production trajectory (0-200 ns) at every frame interval (interval = 1), as specified in the gmx_MMPBSA input file, thereby ensuring comprehensive and unbiased sampling of the conformational space rather than restricting analysis to a subset. 5. Entropy estimation: We clarify that entropy was not explicitly calculated, as quasi-harmonic, interaction entropy, and C2 entropy options were disabled in the gmx_MMPBSA configuration. This is a recognized limitation of MM/PBSA; therefore, the reported binding free energies are primarily enthalpy-driven and suitable for relative ligand ranking. 6. Code availability: The custom HOMO–LUMO script (homo_lumo_nature.py) and input files has been made publicly available (as already addressed in the Data Availability Statement). | 3-7 |
| **Comments 3:** Docking highlights CHEMBL3729403 and CHEMBL3728962 as strongest performers, the MD section additionally describes CHEMBL4299940 as an “excellent candidate,” while MM/PBSA favors CHEMBL3729403 and CHEMBL3729860. The paper would be much stronger if the authors introduced a transparent consensus-ranking or weighted prioritization scheme showing exactly why CHEMBL3729403 and CHEMBL3729860 emerged as the final preferred leads. | **Response 3:** We thank the reviewer for this important suggestion. To improve transparency and consistency across computational results, we have introduced a consensus-based prioritization strategy integrating docking, MD stability, MM/PBSA binding energies, and ADMET properties. This multi-criteria approach explains why CHEMBL3729403 and CHEMBL3729860 were selected as the final lead candidates despite variability across individual methods. The Discussion and Conclusions sections have been revised accordingly. | 27, 29 |
| **Comments 4:** The authors emphasize favorable oral absorption and permeability for CHEMBL3729403 and CHEMBL3729860, which is appropriate, but the manuscript should more clearly discuss the liabilities as well. All compounds were predicted to inhibit major CYP isoenzymes, and the negative QPlogHERG values indicate at least a low but non-negligible cardiotoxicity risk. In addition, CHEMBL3728962 appears weaker from a permeability standpoint despite strong docking-related performance. These caveats deserve more prominence in the Results and Discussion. | **Response 4:** We thank the reviewer for this important observation. We agree that, in addition to highlighting favorable ADMET properties, it is essential to transparently discuss potential liabilities associated with the top-ranked compounds. Accordingly, we have revised the Discussion section to explicitly address predicted CYP inhibition, potential cardiotoxicity risks (QPlogHERG), and permeability limitations observed in certain derivatives (e.g., CHEMBL3728962). These additions provide a more balanced interpretation of the pharmacokinetic and safety profiles and further strengthen the translational relevance of our findings. | 27 |
| **Comments 5:** The disease framing should be tightened. The Introduction moves between thalassemia, PKD, and broader metabolic/oncology relevance, while the abstract is centered on PKD. The primary therapeutic context should be stated more consistently. | **Response 5:** We thank the reviewer for this insightful comment. We agree that the disease framing should be more consistent throughout the manuscript. Accordingly, the Introduction has been revised to clearly position **pyruvate kinase deficiency (PKD) as the primary therapeutic focus**, while thalassemia and other conditions are presented as secondary or emerging applications of PKLR activation. This adjustment ensures alignment with the Abstract and overall study objectives. | 2 |
| **Comments 6:** The manuscript should standardize its terminology for the binding region, since it alternates between “active site,” “binding site,” “allosteric pocket,” and “ligand-binding domain.” | **Response 6:** We thank the reviewer for highlighting this inconsistency. We agree that uniform terminology is essential for clarity. The manuscript has been revised to consistently use the term “PKLR allosteric binding site” when referring to the Mitapivat-binding region. Where appropriate, the term “allosteric pocket” is used as a synonymous descriptor, while ambiguous terms such as “active site” and “ligand-binding domain” have been removed or replaced to avoid confusion with the catalytic site of the enzyme. | All pages |
| **Comments 7:** There appears to be at least one residue-name typo/inconsistency (“Tty400” instead of Tyr400), which should be corrected carefully during revision. | **Response 7:** We thank the reviewer for carefully identifying this typographical inconsistency. We have thoroughly reviewed the manuscript and corrected the residue name from “Tty400” to “Tyr400”. | 22 |
