# Supplementary material for: From structural insight to molecule: Integrative molecular simulations identify candidate pyruvate kinase activators
Source: PLoS One. 2026 Aug 12;21(8):e0352669. doi: 10.1371/journal.pone.0352669 (PMC13466051; doi:10.1371/journal.pone.0352669)
Supplement: Peer review history

# Peer review: Decision Letter 0

**DOI:** 10.1371/journal.pone.0352669.r001

**Authors:** Javed Iqbal

18 Mar 2026

Dear Dr. Alotaiq,

Please submit your revised manuscript by May 02 2026 11:59PM. If you will need more time than this to complete your revisions, please reply to this message or contact the journal office at plosone@plos.org. When you're ready to submit your revision, log on to https://www.editorialmanager.com/pone/ and select the 'Submissions Needing Revision' folder to locate your manuscript file.

- A letter that responds to each point raised by the academic editor and reviewer(s). You should upload this letter as a separate file labeled 'Response to Reviewers'.
- A marked-up copy of your manuscript that highlights changes made to the original version. You should upload this as a separate file labeled 'Revised Manuscript with Track Changes'.
- An unmarked version of your revised paper without tracked changes. You should upload this as a separate file labeled 'Manuscript'.

If you would like to make changes to your financial disclosure, please include your updated statement in your cover letter. Guidelines for resubmitting your figure files are available below the reviewer comments at the end of this letter.

If applicable, we recommend that you deposit your laboratory protocols in protocols.io to enhance the reproducibility of your results. Protocols.io assigns your protocol its own identifier (DOI) so that it can be cited independently in the future. For instructions see: https://journals.plos.org/plosone/s/submission-guidelines#loc-laboratory-protocols. Additionally, PLOS ONE offers an option for publishing peer-reviewed Lab Protocol articles, which describe protocols hosted on protocols.io. Read more information on sharing protocols at https://plos.org/protocols?utm_medium=editorial-email&utm_source=authorletters&utm_campaign=protocols.

We look forward to receiving your revised manuscript.

Kind regards,

Javed Iqbal, PhD

Academic Editor

PLOS One

Journal Requirements:

When submitting your revision, we need you to address these additional requirements.

1. Please ensure that your manuscript meets PLOS ONE's style requirements, including those for file naming. The PLOS ONE style templates can be found at

https://journals.plos.org/plosone/s/file?id=wjVg/PLOSOne_formatting_sample_main_body.pdf and

https://journals.plos.org/plosone/s/file?id=ba62/PLOSOne_formatting_sample_title_authors_affiliations.pdf

2.  Please note that PLOS One has specific guidelines on code sharing for submissions in which author-generated code underpins the findings in the manuscript. In these cases, we expect all author-generated code to be made available without restrictions upon publication of the work. Please review our guidelines at https://journals.plos.org/plosone/s/materials-and-software-sharing#loc-sharing-code and ensure that your code is shared in a way that follows best practice and facilitates reproducibility and reuse.

3. Thank you for stating the following financial disclosure:

“This work was supported and funded by the Deanship of Scientific Research at Imam Mo-hammad Ibn Saud Islamic University (IMSIU) (grant number IMSIU-DDRSP2501).”

Please state what role the funders took in the study. If the funders had no role, please state: "The funders had no role in study design, data collection and analysis, decision to publish, or preparation of the manuscript."

If this statement is not correct you must amend it as needed.

Please include this amended Role of Funder statement in your cover letter; we will change the online submission form on your behalf.

4. We note you have included a table to which you do not refer in the text of your manuscript. Please ensure that you refer to Table 3 in your text; if accepted, production will need this reference to link the reader to the Table.

5. If the reviewer comments include a recommendation to cite specific previously published works, please review and evaluate these publications to determine whether they are relevant and should be cited. There is no requirement to cite these works unless the editor has indicated otherwise.

[Note: HTML markup is below. Please do not edit.]

Reviewers' comments:

Reviewer's Responses to Questions

**Comments to the Author**

1. Is the manuscript technically sound, and do the data support the conclusions?

Reviewer #1: Partly

Reviewer #2: Yes

**********

2. Has the statistical analysis been performed appropriately and rigorously?

Reviewer #1: N/A

Reviewer #2: Yes

**********

3. Have the authors made all data underlying the findings in their manuscript fully available?

The PLOS Data policy

Reviewer #1: Yes

Reviewer #2: Yes

**********

4. Is the manuscript presented in an intelligible fashion and written in standard English?

Reviewer #1: Yes

Reviewer #2: Yes

**********

Reviewer #1: The authors present a computational study aiming to identify next-generation allosteric activators of pyruvate kinase (PKLR), based on the approved drug Mitapivat. The authors employ a multi-step in silico study, including molecular docking, molecular dynamics (MD), and ADMET profiling, to screen a library of Mitapivat derivatives. The study identifies two promising compounds, CHEMBL3729403 and CHEMBL3729860, which exhibit superior binding energies and pharmacokinetic profiles compared to Mitapivat.

The manuscript is well organized but need major revision which are as follows:

1- The authors used HADDOCK to dock small molecules into PKLR. while it is designed primarily for protein–protein or protein–nucleic acid docking and requires ambiguous interaction restraints (AIRs), which are derived from experimental data. the authors did not provide any AIRs. The use of HADDOCK in this context raises concerns about the accuracy and reliability of the reported binding modes and relative affinities as well as conclusions drawn for the top candidates. Redock using a standard small molecule docking tool.

2- The hydrogen bond definition and count are unclear. the manuscript repeatedly refers to carbon-carbon (CC) contacts as "hydrogen bond interactions". CC contacts are non-polar, hydrophobic, or van der Waals interactions, not hydrogen bonds.

3- The abstract claims that CHEMBL3729403 "exhibited the smallest HOMO-LUMO gap". This is incorrect based on the data in Table 4. Mitapivat has a gap of 3.51 eV, while CHEMBL3729403 has a larger gap of 3.59 eV. This error in the abstract must be corrected.

4- provide a reference for allosteric inhibition of PK R-type by phenylalanine

5- It's better to support the results of computational studies with experimental data as (IC50, synthetic condition and in vivo ADME profile)

Reviewer #2: This manuscript presents an interesting and timely computational study aimed at identifying Mitapivat-inspired PKLR activators. The multi-layered workflow; similarity screening, docking, HOMO–LUMO analysis, pharmacophore modeling, 200 ns MD simulations, MM/PBSA, ADMET profiling, and retrosynthetic analysis, is a clear strength. The study also identifies two recurring lead candidates, CHEMBL3729403 and CHEMBL3729860, across several analyses, which gives the work a coherent narrative.

That said, I recommend major revision before the manuscript is suitable for publication.

Major comments

1. The title and parts of the abstract/conclusion imply that the study has “revealed novel pyruvate kinase activators,” but the work is entirely computational. The manuscript itself states that biochemical, cellular, and in vivo validation are still needed to confirm efficacy and safety. The claims should therefore be softened throughout to “candidate,” “putative,” or “predicted” PKLR activators unless experimental validation is added.

2. The overall workflow is described, but key operational details are still missing or too general. The manuscript should clarify: how the 190 compounds were reduced to the final shortlisted set; the exact docking restraints and cluster-selection logic in HADDOCK; whether MD simulations were run once or replicated; how MM/PBSA frames were selected; how entropy was actually estimated; and whether the custom homo_lumo_nature.py script and relevant inputs will be shared. These details are important for a study whose conclusions depend heavily on computational ranking.

3. Docking highlights CHEMBL3729403 and CHEMBL3728962 as strongest performers, the MD section additionally describes CHEMBL4299940 as an “excellent candidate,” while MM/PBSA favors CHEMBL3729403 and CHEMBL3729860. The paper would be much stronger if the authors introduced a transparent consensus-ranking or weighted prioritization scheme showing exactly why CHEMBL3729403 and CHEMBL3729860 emerged as the final preferred leads.

4. The authors emphasize favorable oral absorption and permeability for CHEMBL3729403 and CHEMBL3729860, which is appropriate, but the manuscript should more clearly discuss the liabilities as well. All compounds were predicted to inhibit major CYP isoenzymes, and the negative QPlogHERG values indicate at least a low but non-negligible cardiotoxicity risk. In addition, CHEMBL3728962 appears weaker from a permeability standpoint despite strong docking-related performance. These caveats deserve more prominence in the Results and Discussion.

Minor comments

- The disease framing should be tightened. The Introduction moves between thalassemia, PKD, and broader metabolic/oncology relevance, while the abstract is centered on PKD. The primary therapeutic context should be stated more consistently.

- The manuscript should standardize its terminology for the binding region, since it alternates between “active site,” “binding site,” “allosteric pocket,” and “ligand-binding domain.”

- There appears to be at least one residue-name typo/inconsistency (“Tty400” instead of Tyr400), which should be corrected carefully during revision.

The study has a solid computational foundation and a potentially publishable core, but it currently overstates its biological conclusions and needs a clearer reproducibility framework, a stronger justification for final lead prioritization, and a more balanced treatment of ADMET and validation limitations. I therefore recommend major revision.

**********

what does this mean?). If published, this will include your full peer review and any attached files.

If you choose “no”, your identity will remain anonymous but your review may still be made public.

**Do you want your identity to be public for this peer review?**  For information about this choice, including consent withdrawal, please see our Privacy Policy

Reviewer #1: No

Reviewer #2: No

**********

[NOTE: If reviewer comments were submitted as an attachment file, they will be attached to this email and accessible via the submission site. Please log into your account, locate the manuscript record, and check for the action link "View Attachments". If this link does not appear, there are no attachment files.]

To ensure your figures meet our technical requirements, please review our figure guidelines: https://journals.plos.org/plosone/s/figures

You may also use PLOS’s free figure tool, NAAS, to help you prepare publication quality figures: https://journals.plos.org/plosone/s/figures#loc-tools-for-figure-preparation.

NAAS will assess whether your figures meet our technical requirements by comparing each figure against our figure specifications.

# Author comment: Author response to Decision Letter 1

**DOI:** 10.1371/journal.pone.0352669.r002

31 Mar 2026

EDITOR COMMENTS:

Comments 1: Please ensure that your manuscript meets PLOS ONE's style requirements, including those for file naming. The PLOS ONE style templates can be found at

https://journals.plos.org/plosone/s/file?id=wjVg/PLOSOne_formatting_sample_main_body.pdf and

https://journals.plos.org/plosone/s/file?id=ba62/PLOSOne_formatting_sample_title_authors_affiliations.pdf

Response 1: Thank you for your guidance. We have carefully revised the manuscript to ensure full compliance with PLOS ONE’s style requirements. Specifically, we have adjusted the formatting of the main text, title page, author names, and affiliations in accordance with the official PLOS ONE templates provided. In addition, we have reviewed and updated file naming conventions, figure and table formatting, section organization, and overall manuscript structure to align with PLOS ONE guidelines.

Comments 2: Please note that PLOS One has specific guidelines on code sharing for submissions in which author-generated code underpins the findings in the manuscript. In these cases, we expect all author-generated code to be made available without restrictions upon publication of the work. Please review our guidelines at https://journals.plos.org/plosone/s/materials-and-software-sharing#loc-sharing-code and ensure that your code is shared in a way that follows best practice and facilitates reproducibility and reuse.

Response 2: In response, we have ensured that all custom scripts used in this study (including the DFT-based HOMO–LUMO analysis workflow) are made publicly accessible without restriction. The code has been deposited in a publicly available repository (GitHub), and the corresponding link has been included in the revised manuscript under the Data Availability section.

Comments 3: Thank you for stating the following financial disclosure: “This work was supported and funded by the Deanship of Scientific Research at Imam Mohammad Ibn Saud Islamic University (IMSIU) (grant number IMSIU-DDRSP2501).”

Please state what role the funders took in the study. If the funders had no role, please state: "The funders had no role in study design, data collection and analysis, decision to publish, or preparation of the manuscript." If this statement is not correct you must amend it as needed. Please include this amended Role of Funder statement in your cover letter; we will change the online submission form on your behalf.

Response 3: Thank you for your comment. We would like to inform you that the grant number has been corrected to IMSIU-DDRSP2601 (due to the year of publication - 2026) in the revised manuscript. In addition, we have included the required statement regarding the role of the funder. The revised Financial Disclosure section now reads:

“This work was supported and funded by the Deanship of Scientific Research at Imam Mohammad Ibn Saud Islamic University (IMSIU) (grant number IMSIU-DDRSP2601). The funders had no role in study design, data collection and analysis, decision to publish, or preparation of the manuscript.”

Comments 4: We note you have included a table to which you do not refer in the text of your manuscript. Please ensure that you refer to Table 3 in your text; if accepted, production will need this reference to link the reader to the Table.

Response 4: Thank you for pointing this out. We have carefully revised the manuscript to ensure that Table 3 is now appropriately cited and discussed in the main text. The relevant section has been updated to clearly refer to Table 3, allowing proper linkage between the text and the presented data.

Comments 5: If the reviewer comments include a recommendation to cite specific previously published works, please review and evaluate these publications to determine whether they are relevant and should be cited. There is no requirement to cite these works unless the editor has indicated otherwise.

Response 5: Thank you for your guidance. We have carefully reviewed the reviewer comments and confirm that no specific references were suggested for inclusion. Therefore, no additional citations were required or added to the manuscript in this regard.

REVIEWER 1 COMMENTS

Comments 1: The authors used HADDOCK to dock small molecules into PKLR. while it is designed primarily for protein–protein or protein–nucleic acid docking and requires ambiguous interaction restraints (AIRs), which are derived from experimental data. the authors did not provide any AIRs. The use of HADDOCK in this context raises concerns about the accuracy and reliability of the reported binding modes and relative affinities as well as conclusions drawn for the top candidates. Redock using a standard small molecule docking tool.

Response 1: We thank the reviewer for this important comment regarding the use of HADDOCK for small-molecule docking.

First, we would like to clarify that blind docking was not performed in this study. Instead, docking simulations were conducted using experimentally validated active-site information derived from the crystal structure of PKLR in complex with Mitapivat (PDB ID: 8XFD). The binding site and key residues were identified using PDBsum, ensuring that docking was restricted to a biologically relevant allosteric pocket.

The following active-site residues were explicitly defined and used as input restraints in HADDOCK:

36, 37, 40, 321, 363, 364, 399, 400, and 404. These residues have now been clearly added to the revised Materials and Methods section and are also illustrated in Supplementary Figure S1.

Furthermore, we employed HADDOCK in ligand-docking mode, which is specifically adapted for protein–small molecule interactions. Although HADDOCK is widely known for protein–protein docking, it has been successfully applied to ligand docking when structural information is available, as in our case.

To validate the docking protocol, we performed re-docking of the co-crystallized ligand (Mitapivat). The results demonstrated high consistency with the experimental binding pose, including preservation of key interactions such as hydrogen bonding with Tyr400, and yielded an RMSD of 0.1 ± 0.1 Å, indicating excellent agreement with the crystallographic structure.

This successful re-docking validation confirms the reliability of our docking setup. Therefore, we believe that the use of additional docking tools is not strictly necessary, as the current protocol already demonstrates high accuracy, reproducibility, and agreement with experimental data.

We have clarified these points in the revised manuscript to improve methodological transparency and rigor.

Comments 2: The hydrogen bond definition and count are unclear. the manuscript repeatedly refers to carbon-carbon (CC) contacts as "hydrogen bond interactions". CC contacts are non-polar, hydrophobic, or van der Waals interactions, not hydrogen bonds.

Response 2: We thank the reviewer for this important clarification. We fully agree that carbon–carbon (CC) contacts do not represent hydrogen bonds, but rather correspond to non-polar, hydrophobic, or van der Waals interactions.

In the original manuscript, the term “hydrogen bond interactions” was inadvertently used as a general descriptor for all intermolecular contacts categorized by atom pair types. We acknowledge that this terminology was inaccurate and may lead to confusion.

To address this, we have carefully revised the manuscript to:

1. Replace the term “hydrogen bond interactions” with “intermolecular interactions” where appropriate.

2. Clearly distinguish between true hydrogen bonds (e.g., involving O and N atoms) and non-polar contacts (e.g., CC interactions).

3. Update the corresponding text in the Results section to reflect the correct chemical interpretation.

Comments 3: The abstract claims that CHEMBL3729403 "exhibited the smallest HOMO-LUMO gap". This is incorrect based on the data in Table 4. Mitapivat has a gap of 3.51 eV, while CHEMBL3729403 has a larger gap of 3.59 eV. This error in the abstract must be corrected.

Response 3: We thank the reviewer for carefully identifying this inconsistency. We acknowledge that the statement in the original abstract incorrectly described CHEMBL3729403 as having the smallest HOMO–LUMO gap. Based on the data presented in Table 4, Mitapivat exhibits the smallest gap (3.51 eV), while CHEMBL3729403 shows a slightly larger value (3.59 eV). We have corrected the abstract accordingly to accurately reflect the results and avoid misinterpretation. The revised text now appropriately describes the electronic properties of CHEMBL3729403 without overstating its HOMO–LUMO gap relative to Mitapivat.

Comments 4: Provide a reference for allosteric inhibition of PKLR-type by phenylalanine

Response 4: We have added appropriate references that clearly demonstrate the role of phenylalanine as a negative allosteric regulator of pyruvate kinase. These references are now incorporated into the revised manuscript to strengthen the scientific basis of our methodology.

Comments 5: It's better to support the results of computational studies with experimental data as (IC50, synthetic condition and in vivo ADME profile)

Response 5: We thank the reviewer for this important suggestion. We fully agree that experimental validation, including IC₅₀ determination, synthetic procedures, and in vivo ADME profiling, is essential to confirm the pharmacological potential of the proposed compounds.

However, the primary objective of the present study was to perform a comprehensive multiscale in silico investigation, integrating molecular docking, pharmacophore modeling, MD simulations, MM/PBSA free energy calculations, ADMET prediction, and retrosynthetic analysis to prioritize promising Mitapivat derivatives. As such, experimental validation was beyond the scope of this work.

To address this limitation, we have explicitly incorporated the reviewer’s suggestion into the “Limitations and future works” section, where we now emphasize the necessity of future experimental studies, including biochemical assays (e.g., IC₅₀), synthetic validation, and in vivo pharmacokinetic evaluation.

REVIWER 2 COMMENTS

Comments 1: The title and parts of the abstract/conclusion imply that the study has “revealed novel pyruvate kinase activators,” but the work is entirely computational. The manuscript itself states that biochemical, cellular, and in vivo validation are still needed to confirm efficacy and safety. The claims should therefore be softened throughout to “candidate,” “putative,” or “predicted” PKLR activators unless experimental validation is added.

Response 1: We thank the reviewer for this important and valid comment. We agree that, as this study is entirely computational, the terminology should be carefully framed to avoid overstating the findings. Accordingly, we have revised the title, abstract, and conclusions to replace definitive terms such as “activators” with more appropriate language including “candidate,” “putative,” and “predicted” PKLR activators. These changes ensure that the claims are fully aligned with the in silico nature of the work while maintaining scientific accuracy and rigor.

Comments 2: The overall workflow is described, but key operational details are still missing or too general. The manuscript should clarify: how the 190 compounds were reduced to the final shortlisted set; the exact docking restraints and cluster-selection logic in HADDOCK; whether MD simulations were run once or replicated; how MM/PBSA frames were selected; how entropy was actually estimated; and whether the custom homo_lumo_nature.py script and relevant inputs will be shared. These details are important for a study whose conclusions depend heavily on computational ranking.

Response 2: We thank the reviewer for this constructive comment, highlighting the need for greater methodological transparency. In response, we have clarified several key operational aspects of the workflow to improve reproducibility and rigor:

1. Compound selection: We now explicitly state that the initial pool of ~400 compounds retrieved from SwissSimilarity was filtered using a similarity threshold (≥ 0.90), resulting in 190 compounds subjected to docking.

2. Docking restraints and selection: The active-site residues derived from the co-crystallized structure (PDB ID: 8XFD) via PDBsum were used as restraints in HADDOCK ligand-docking mode. Cluster selection was based on both cluster size (population) and HADDOCK score, with the most populated and energetically favorable cluster selected.

3. MD simulations: We clarify that a single 200 ns production run per system was performed following proper equilibration.

4. MM/PBSA frame selection: Frames were extracted from the full production trajectory (0-200 ns) at every frame interval (interval = 1), as specified in the gmx_MMPBSA input file, thereby ensuring comprehensive and unbiased sampling of the conformational space rather than restricting analysis to a subset.

5. Entropy estimation: We clarify that entropy was not explicitly calculated, as quasi-harmonic, interaction entropy, and C2 entropy options were disabled in the gmx_MMPBSA configuration. This is a recognized limitation of MM/PBSA; therefore, the reported binding free energies are primarily enthalpy-driven and suitable for relative ligand ranking.

6. Code availability: The custom HOMO–LUMO script (homo_lumo_nature.py) and input files has been made publicly available (as already addressed in the Data Availability Statement).

Comments 3: Docking highlights CHEMBL3729403 and CHEMBL3728962 as strongest performers, the MD section additionally describes CHEMBL4299940 as an “excellent candidate,” while MM/PBSA favors CHEMBL3729403 and CHEMBL3729860. The paper would be much stronger if the authors introduced a transparent consensus-ranking or weighted prioritization scheme showing exactly why CHEMBL3729403 and CHEMBL3729860 emerged as the final preferred leads.

Response 3: We thank the reviewer for this important suggestion. To improve transparency and consistency across computational results, we have introduced a consensus-based prioritization strategy integrating docking, MD stability, MM/PBSA binding energies, and ADMET properties. This multi-criteria approach explains why CHEMBL3729403 and CHEMBL3729860 were selected as the final lead candidates despite variability across individual methods. The Discussion and Conclusions sections have been revised accordingly.

Comments 4: The authors emphasize favorable oral absorption and permeability for CHEMBL3729403 and CHEMBL3729860, which is appropriate, but the manuscript should more clearly discuss the liabilities as well. All compounds were predicted to inhibit major CYP isoenzymes, and the negative QPlogHERG values indicate at least a low but non-negligible cardiotoxicity risk. In addition, CHEMBL3728962 appears weaker from a permeability standpoint despite strong docking-related performance. These caveats deserve more prominence in the Results and Discussion.

Response 4: We thank the reviewer for this important observation. We agree that, in addition to highlighting favorable ADMET properties, it is essential to transparently discuss potential liabilities associated with the top-ranked compounds. Accordingly, we have revised the Discussion section to explicitly address predicted CYP inhibition, potential cardiotoxicity risks (QPlogHERG), and permeability limitations observed in certain derivatives (e.g., CHEMBL3728962). These additions provide a more balanced interpretation of the pharmacokinetic and safety profiles and further strengthen the translational relevance of our findings.

Comments 5: The disease framing should be tightened. The Introduction moves between thalassemia, PKD, and broader metabolic/oncology relevance, while the abstract is centered on PKD. The primary therapeutic context should be stated more consistently.

Response 5: We thank the reviewer for this insightful comment. We agree that the disease framing should be more consistent throughout the manuscript. Accordingly, the Introduction has been revised to clearly position pyruvate kinase deficiency (PKD) as the primary therapeutic focus, while thalassemia and other conditions are presented as secondary or emerging applications of PKLR activation. This adjustment ensures alignment with the Abstract and overall study o

Attachment Submitted filename: [PLOS One] - Reply to Reviewers.docx

# Peer review: Decision Letter 1

**DOI:** 10.1371/journal.pone.0352669.r003

**Authors:** Javed Iqbal

26 Apr 2026

Dear Dr. Alotaiq,

Thank you for submitting your manuscript to PLOS ONE. After careful consideration, we feel that it has merit but does not fully meet PLOS ONE’s publication criteria as it currently stands. Therefore, we invite you to submit a revised version of the manuscript that addresses the points raised during the review process.

The revised version has been carefully evaluated. While some improvements have been made, it is evident that several key concerns raised during the initial review by Reviewer 1 have not been adequately addressed or justified.

In particular:

- The methodological concern regarding the use of HADDOCK for small-molecule docking remains insufficiently resolved. The justification provided does not adequately support its applicability in the absence of appropriate restraints, and the reliability of the reported binding modes is still questionable.
- The issue related to the incorrect classification of interaction types, specifically the misinterpretation of carbon–carbon contacts as hydrogen bonds, has not been fully corrected throughout the manuscript.
- The inconsistency in HOMO–LUMO gap interpretation persists, and the corresponding statements require careful revision to align with the reported data.
- The manuscript still lacks appropriate literature support for certain mechanistic claims, including allosteric inhibition by phenylalanine.
- Importantly, the study continues to rely entirely on computational findings without sufficient discussion of limitations or experimental validation, which weakens the strength of the conclusions.

Please submit your revised manuscript by Jun 10 2026 11:59PM. If you will need more time than this to complete your revisions, please reply to this message or contact the journal office at plosone@plos.org. When you're ready to submit your revision, log on to https://www.editorialmanager.com/pone/ and select the 'Submissions Needing Revision' folder to locate your manuscript file.

- A letter that responds to each point raised by the academic editor and reviewer(s). You should upload this letter as a separate file labeled 'Response to Reviewers'.
- A marked-up copy of your manuscript that highlights changes made to the original version. You should upload this as a separate file labeled 'Revised Manuscript with Track Changes'.
- An unmarked version of your revised paper without tracked changes. You should upload this as a separate file labeled 'Manuscript'.

If you would like to make changes to your financial disclosure, please include your updated statement in your cover letter. Guidelines for resubmitting your figure files are available below the reviewer comments at the end of this letter.

If applicable, we recommend that you deposit your laboratory protocols in protocols.io to enhance the reproducibility of your results. Protocols.io assigns your protocol its own identifier (DOI) so that it can be cited independently in the future. For instructions see: https://journals.plos.org/plosone/s/submission-guidelines#loc-laboratory-protocols. Additionally, PLOS ONE offers an option for publishing peer-reviewed Lab Protocol articles, which describe protocols hosted on protocols.io. Read more information on sharing protocols at https://plos.org/protocols?utm_medium=editorial-email&utm_source=authorletters&utm_campaign=protocols.

As the corresponding author, your ORCID iD is verified in the submission system and will appear in the published article. PLOS supports the use of ORCID, and we encourage all coauthors to register for an ORCID iD and use it as well. Please encourage your coauthors to verify their ORCID iD within the submission system before final acceptance, as unverified ORCID iDs will not appear in the published article. *Only*  the individual author can complete the verification step; PLOS staff *cannot*  verify ORCID iDs on behalf of authors.

We look forward to receiving your revised manuscript.

Kind regards,

Javed Iqbal, PhD

Academic Editor

PLOS One

Journal Requirements:

If the reviewer comments include a recommendation to cite specific previously published works, please review and evaluate these publications to determine whether they are relevant and should be cited. There is no requirement to cite these works unless the editor has indicated otherwise.

[Note: HTML markup is below. Please do not edit.]

Reviewers' comments:

Reviewer's Responses to Questions

**Comments to the Author**

Reviewer #2: All comments have been addressed

Reviewer #3: (No Response)

**********

2. Is the manuscript technically sound, and do the data support the conclusions?

Reviewer #2: Yes

Reviewer #3: (No Response)

**********

3. Has the statistical analysis been performed appropriately and rigorously?

Reviewer #2: Yes

Reviewer #3: (No Response)

**********

4. Have the authors made all data underlying the findings in their manuscript fully available?

The PLOS Data policy

Reviewer #2: Yes

Reviewer #3: (No Response)

**********

5. Is the manuscript presented in an intelligible fashion and written in standard English?

Reviewer #2: Yes

Reviewer #3: (No Response)

**********

Reviewer #2: The authors have fully addressed all of my previous concerns. The revisions have strengthened the technical depth and clarity of the manuscript. The methodology is now robustly described, and the conclusions are well-supported by the data provided. I have no further comments and recommend the paper for acceptance in its current form.

Reviewer #3: I read the manuscript with interest.

The work is exciting, but some points should be fixed before publication in PLOS One.

1. The abstract should be improved according to the results.

2. The introduction should be improved by adding recent references. These references should be added to improve it.

https://doi.org/10.3390/molecules24091714

https://doi.org/10.1016/j.molstruc.2019.02.071

https://doi.org/10.1007/s10753-025-02289-2

https://doi.org/10.2174/0113816128393399251021101651

https://doi.org/10.1080/13813455.2026.2628187

https://doi.org/10.3390/antiox15010109

3. Justify the usage of the functional and basis set (B3LYP level with the 6-311+G (d, p)) used in the calculations.

4. The quality of some figures should be improved.

5. What is the motivation behind the selection of the protein PDB: 8XFD?

6. Finally, the conclusion section should be expanded to provide a more comprehensive summary. It should include the authors' perspectives on future research directions in this field.

**********

what does this mean?). If published, this will include your full peer review and any attached files.

If you choose “no”, your identity will remain anonymous but your review may still be made public.

**Do you want your identity to be public for this peer review?**  For information about this choice, including consent withdrawal, please see our Privacy Policy

Reviewer #2: No

Reviewer #3: No

**********

[NOTE: If reviewer comments were submitted as an attachment file, they will be attached to this email and accessible via the submission site. Please log into your account, locate the manuscript record, and check for the action link "View Attachments". If this link does not appear, there are no attachment files.]

To ensure your figures meet our technical requirements, please review our figure guidelines: https://journals.plos.org/plosone/s/figures

You may also use PLOS’s free figure tool, NAAS, to help you prepare publication quality figures: https://journals.plos.org/plosone/s/figures#loc-tools-for-figure-preparation.

NAAS will assess whether your figures meet our technical requirements by comparing each figure against our figure specifications.

# Author comment: Author response to Decision Letter 2

**DOI:** 10.1371/journal.pone.0352669.r004

28 Apr 2026

REVIEWER 1 COMMENTS (please see the response to reviewer for the detailed revisions and pages)

Comments 1: The methodological concern regarding the use of HADDOCK for small-molecule docking remains insufficiently resolved. The justification provided does not adequately support its applicability in the absence of appropriate restraints, and the reliability of the reported binding modes is still questionable.

Response 1: We thank the reviewer for raising this important methodological concern. We acknowledge that HADDOCK is primarily developed for biomolecular docking; however, its application to small-molecule docking in the present study was carefully justified and implemented using a data-driven, restraint-guided approach based on experimentally validated binding-site residues derived from the co-crystallized Mitapivat–PKLR complex (PDB ID: 8XFD). Specifically, active residues (His39, Tyr400, and surrounding pocket residues) were defined as ambiguous interaction restraints to restrict the conformational search space to biologically relevant regions. Importantly, the reliability of this protocol was validated through successful re-docking of Mitapivat, reproducing the crystallographic binding pose with very low RMSD (~0.1 Å) and preserving key interactions, thereby demonstrating the accuracy of the docking setup even in the absence of ligand-specific restraints. Furthermore, docking predictions were not interpreted in isolation but were consistently supported by independent MD simulations, MM/PBSA free energy calculations, and interaction analyses, all of which converged on the same binding modes and key residues. Therefore, we believe that the combination of restraint-guided docking, experimental validation via re-docking, and multi-method consensus provides sufficient confidence in the reliability of the reported binding poses. The related method section was revised accordingly.

Comments 2: The issue related to the incorrect classification of interaction types, specifically the misinterpretation of carbon–carbon contacts as hydrogen bonds, has not been fully corrected throughout the manuscript.

Response 2: We thank the reviewer for highlighting this important issue. We fully acknowledge that earlier versions of the manuscript contained imprecise wording that could be interpreted as conflating carbon–carbon contacts with hydrogen bonding interactions. This has now been systematically corrected throughout the manuscript. In particular, we explicitly clarify that carbon–carbon (CC) interactions represent hydrophobic and van der Waals contacts only and do not correspond to hydrogen bonds, while true hydrogen bonding is restricted to interactions involving electronegative atoms such as oxygen and nitrogen (e.g., O–O, O–N, N–O pairs). We have carefully revised the text to ensure consistent and accurate classification of interaction types and removed any ambiguous phrasing. Importantly, all interpretations of binding mechanisms have been updated to reflect this corrected framework, without altering the overall conclusions of the study. These revisions ensure that the interaction analysis is now chemically rigorous and aligned with standard definitions in molecular recognition studies.

Comments 3: The inconsistency in HOMO–LUMO gap interpretation persists, and the corresponding statements require careful revision to align with the reported data.

Response 3: We thank the reviewer for pointing out the inconsistency in the interpretation of HOMO–LUMO gaps. We have now carefully revised the manuscript to ensure that all statements are fully consistent with the reported data and standard quantum chemical interpretation. Specifically, we avoid overgeneralization by clarifying that variations in HOMO–LUMO gap within the narrow range observed (3.51–3.90 eV) indicate only modest differences in electronic stability and reactivity, rather than definitive superiority of any compound. Larger gaps are now described as reflecting slightly increased kinetic stability and lower chemical reactivity, while comparable gaps are interpreted as similar electronic behavior, without implying enhanced biological activity. Importantly, we explicitly state that FMO results are supportive and not determinative, and must be interpreted alongside docking, MD, and MM/PBSA analyses.

Comments 4: The manuscript still lacks appropriate literature support for certain mechanistic claims, including allosteric inhibition by phenylalanine.

Response 4: We appreciate the concern. We have revised the paragraph to cite primary literature for phenylalanine’s effect on PK. In particular, phenylalanine is known to inhibit all major PK isoforms (Uyeda, 2013), while more recent experimental evidence (Nilsson et al., 2025) demonstrates that phenylalanine does not significantly interfere with binding of allosteric activators at the PKLR site. Thus, in contrast to potent activators such as Mitapivat, phenylalanine’s effect on PKLR is relatively modest and context-dependent. Our computational results, which show weak binding of phenylalanine compared to Mitapivat and its derivatives, are fully consistent with these experimentally reported observations. The discussion section was revised accordingly.

Comments 5: Importantly, the study continues to rely entirely on computational findings without sufficient discussion of limitations or experimental validation, which weakens the strength of the conclusions.

Response 5: We appreciate this important comment and agree that relying solely on computational evidence can limit the strength of the conclusions if not properly contextualized. In response, we have substantially revised the Limitations and Future Works section to more explicitly acknowledge the constraints of docking, MD simulations, MM/PBSA, and in silico ADMET predictions, and to clearly position our findings as hypothesis-generating rather than confirmatory. We now explicitly outline a stepwise experimental validation framework, including compound synthesis, in vitro enzymatic assays (EC₅₀/activation studies), structural validation (X-ray/cryo-EM), red blood cell functional assays, and in vivo pharmacokinetics, to bridge computational predictions with biological evidence. These additions clarify that our study provides a rational prioritization strategy for candidate PKLR activators, while emphasizing that definitive validation requires experimental investigation.

Attachment Submitted filename: [PLOS One] - Reply to Reviewers - 2nd Round.pdf

# Peer review: Decision Letter 2

**DOI:** 10.1371/journal.pone.0352669.r005

**Authors:** Javed Iqbal

20 May 2026

Dear Dr. Alotaiq,

Thank you for submitting your manuscript to PLOS ONE. After careful consideration, we feel that it has merit but does not fully meet PLOS ONE’s publication criteria as it currently stands. Therefore, we invite you to submit a revised version of the manuscript that addresses the points raised during the review process.

One of the reviewers has indicated that his comments have not been addressed in the current revision. Therefore, a more thorough revision is necessary to ensure that all reviewers' concerns are properly addressed in detail.

You are requested to carefully revise the manuscript, provide a point-by-point response to all reviewer comments, and clearly indicate the changes made in the revised version.

Please submit your revised manuscript by Jul 04 2026 11:59PM. If you will need more time than this to complete your revisions, please reply to this message or contact the journal office at plosone@plos.org. When you're ready to submit your revision, log on to https://www.editorialmanager.com/pone/ and select the 'Submissions Needing Revision' folder to locate your manuscript file.

- A letter that responds to each point raised by the academic editor and reviewer(s). You should upload this letter as a separate file labeled 'Response to Reviewers'.
- A marked-up copy of your manuscript that highlights changes made to the original version. You should upload this as a separate file labeled 'Revised Manuscript with Track Changes'.
- An unmarked version of your revised paper without tracked changes. You should upload this as a separate file labeled 'Manuscript'.

If you would like to make changes to your financial disclosure, please include your updated statement in your cover letter. Guidelines for resubmitting your figure files are available below the reviewer comments at the end of this letter.

If applicable, we recommend that you deposit your laboratory protocols in protocols.io to enhance the reproducibility of your results. Protocols.io assigns your protocol its own identifier (DOI) so that it can be cited independently in the future. For instructions see: https://journals.plos.org/plosone/s/submission-guidelines#loc-laboratory-protocols. Additionally, PLOS ONE offers an option for publishing peer-reviewed Lab Protocol articles, which describe protocols hosted on protocols.io. Read more information on sharing protocols at https://plos.org/protocols?utm_medium=editorial-email&utm_source=authorletters&utm_campaign=protocols.

As the corresponding author, your ORCID iD is verified in the submission system and will appear in the published article. PLOS supports the use of ORCID, and we encourage all coauthors to register for an ORCID iD and use it as well. Please encourage your coauthors to verify their ORCID iD within the submission system before final acceptance, as unverified ORCID iDs will not appear in the published article. *Only*  the individual author can complete the verification step; PLOS staff *cannot*  verify ORCID iDs on behalf of authors.

We look forward to receiving your revised manuscript.

Kind regards,

Javed Iqbal, PhD

Academic Editor

PLOS One

Journal Requirements:

If the reviewer comments include a recommendation to cite specific previously published works, please review and evaluate these publications to determine whether they are relevant and should be cited. There is no requirement to cite these works unless the editor has indicated otherwise.

[Note: HTML markup is below. Please do not edit.]

Reviewers' comments:

Reviewer's Responses to Questions

**Comments to the Author**

Reviewer #3: (No Response)

**********

2. Is the manuscript technically sound, and do the data support the conclusions?

Reviewer #3: (No Response)

**********

3. Has the statistical analysis been performed appropriately and rigorously?

Reviewer #3: (No Response)

**********

4. Have the authors made all data underlying the findings in their manuscript fully available?

The PLOS Data policy

Reviewer #3: (No Response)

**********

5. Is the manuscript presented in an intelligible fashion and written in standard English?

Reviewer #3: (No Response)

**********

Reviewer #3: Dear editor,

I didn't find any response to my comments. Have you forwarded them to the authors or not?

**********

what does this mean?). If published, this will include your full peer review and any attached files.

If you choose “no”, your identity will remain anonymous but your review may still be made public.

**Do you want your identity to be public for this peer review?**  For information about this choice, including consent withdrawal, please see our Privacy Policy

Reviewer #3: No

**********

[NOTE: If reviewer comments were submitted as an attachment file, they will be attached to this email and accessible via the submission site. Please log into your account, locate the manuscript record, and check for the action link "View Attachments". If this link does not appear, there are no attachment files.]

To ensure your figures meet our technical requirements, please review our figure guidelines: https://journals.plos.org/plosone/s/figures

You may also use PLOS’s free figure tool, NAAS, to help you prepare publication quality figures: https://journals.plos.org/plosone/s/figures#loc-tools-for-figure-preparation.

NAAS will assess whether your figures meet our technical requirements by comparing each figure against our figure specifications.

# Author comment: Author response to Decision Letter 3

**DOI:** 10.1371/journal.pone.0352669.r006

2 Jun 2026

The response is attached.

Attachment Submitted filename: [PLOS One] - Reply to Reviewers.pdf

# Editor report: Decision Letter 3

**DOI:** 10.1371/journal.pone.0352669.r007

**Authors:** Javed Iqbal

3 Jun 2026

PONE-D-25-46615R3

From structural insight to molecule: Integrative molecular simulations identify candidate pyruvate kinase activators

PLOS One

Dear Dr. Alotaiq,

Thank you for submitting your manuscript to PLOS ONE. After careful consideration, we feel that it has merit but does not fully meet PLOS ONE’s publication criteria as it currently stands. Therefore, we invite you to submit a revised version of the manuscript that addresses the points raised during the review process.

The following are the Reviewer 3 comments which were not addressed during the last round of revision.

1. The abstract should be improved according to the results.

2. The introduction should be improved by adding recent references. These references should be added to improve it.

https://doi.org/10.3390/molecules24091714

https://doi.org/10.1016/j.molstruc.2019.02.071

https://doi.org/10.1007/s10753-025-02289-2

https://doi.org/10.2174/0113816128393399251021101651

https://doi.org/10.1080/13813455.2026.2628187

https://doi.org/10.3390/antiox15010109

3. Justify the usage of the functional and basis set (B3LYP level with the 6-311+G (d, p)) used in the calculations.

4. The quality of some figures should be improved.

5. What is the motivation behind the selection of the protein PDB: 8XFD?

6. Finally, the conclusion section should be expanded to provide a more comprehensive summary. It should include the authors' perspectives on future research directions in this field.

Please submit your revised manuscript by Jul 18 2026 11:59PM. If you will need more time than this to complete your revisions, please reply to this message or contact the journal office at plosone@plos.org. When you're ready to submit your revision, log on to https://www.editorialmanager.com/pone/ and select the 'Submissions Needing Revision' folder to locate your manuscript file.

Please include the following items when submitting your revised manuscript:

- A letter that responds to each point raised by the academic editor and reviewer(s). You should upload this letter as a separate file labeled 'Response to Reviewers'.
- A marked-up copy of your manuscript that highlights changes made to the original version. You should upload this as a separate file labeled 'Revised Manuscript with Track Changes'.
- An unmarked version of your revised paper without tracked changes. You should upload this as a separate file labeled 'Manuscript'.

If you would like to make changes to your financial disclosure, please include your updated statement in your cover letter. Guidelines for resubmitting your figure files are available below the reviewer comments at the end of this letter.

If applicable, we recommend that you deposit your laboratory protocols in protocols.io to enhance the reproducibility of your results. Protocols.io assigns your protocol its own identifier (DOI) so that it can be cited independently in the future. For instructions see: https://journals.plos.org/plosone/s/submission-guidelines#loc-laboratory-protocols. Additionally, PLOS ONE offers an option for publishing peer-reviewed Lab Protocol articles, which describe protocols hosted on protocols.io. Read more information on sharing protocols at https://plos.org/protocols?utm_medium=editorial-email&utm_source=authorletters&utm_campaign=protocols.

As the corresponding author, your ORCID iD is verified in the submission system and will appear in the published article. PLOS supports the use of ORCID, and we encourage all coauthors to register for an ORCID iD and use it as well. Please encourage your coauthors to verify their ORCID iD within the submission system before final acceptance, as unverified ORCID iDs will not appear in the published article. *Only*  the individual author can complete the verification step; PLOS staff *cannot*  verify ORCID iDs on behalf of authors.

We look forward to receiving your revised manuscript.

Kind regards,

Javed Iqbal, PhD

Academic Editor

PLOS One

**Journal Requirements:**

If the reviewer comments include a recommendation to cite specific previously published works, please review and evaluate these publications to determine whether they are relevant and should be cited. There is no requirement to cite these works unless the editor has indicated otherwise.

[Note: HTML markup is below. Please do not edit.]

[NOTE: If reviewer comments were submitted as an attachment file, they will be attached to this email and accessible via the submission site. Please log into your account, locate the manuscript record, and check for the action link "View Attachments". If this link does not appear, there are no attachment files.]

To ensure your figures meet our technical requirements, please review our figure guidelines: https://journals.plos.org/plosone/s/figures

You may also use PLOS’s free figure tool, NAAS, to help you prepare publication quality figures: https://journals.plos.org/plosone/s/figures#loc-tools-for-figure-preparation.

NAAS will assess whether your figures meet our technical requirements by comparing each figure against our figure specifications.

# Author comment: Author response to Decision Letter 4

**DOI:** 10.1371/journal.pone.0352669.r008

7 Jun 2026

Dear Reviewer #3,

Please kindly find our response to your comments in the "Reply to Reviewers" file.

Thank you very much.

Regards,

Dr. Alotaiq

Attachment Submitted filename: [PLOS_One]_-_Reply_to_Reviewers_auresp_4.pdf

# Peer review: Decision Letter 4

**DOI:** 10.1371/journal.pone.0352669.r009

**Authors:** Javed Iqbal

15 Jun 2026

From structural insight to molecule: Integrative molecular simulations identify candidate pyruvate kinase activators

PONE-D-25-46615R4

Dear Author,

We’re pleased to inform you that your manuscript has been judged scientifically suitable for publication and will be formally accepted for publication once it meets all outstanding technical requirements.

Within one week, you’ll receive an e-mail detailing the required amendments. When these have been addressed, you’ll receive a formal acceptance letter and your manuscript will be scheduled for publication.

An invoice will be generated when your article is formally accepted. Please note, if your institution has a publishing partnership with PLOS and your article meets the relevant criteria, all or part of your publication costs will be covered. Please make sure your user information is up-to-date by logging into Editorial Manager at Editorial Manager® and clicking the ‘Update My Information' link at the top of the page. For questions related to billing, please contact billing support.

If your institution or institutions have a press office, please notify them about your upcoming paper to help maximize its impact. If they’ll be preparing press materials, please inform our press team as soon as possible -- no later than 48 hours after receiving the formal acceptance. Your manuscript will remain under strict press embargo until 2 pm Eastern Time on the date of publication. For more information, please contact onepress@plos.org.

Kind regards,

Javed Iqbal, PhD

Academic Editor

PLOS One

Additional Editor Comments (optional):

Reviewers' comments:

Reviewer's Responses to Questions

**Comments to the Author**

Reviewer #3: All comments have been addressed

**********

2. Is the manuscript technically sound, and do the data support the conclusions?

Reviewer #3: Yes

**********

3. Has the statistical analysis been performed appropriately and rigorously?

Reviewer #3: N/A

**********

4. Have the authors made all data underlying the findings in their manuscript fully available?

The PLOS Data policy

Reviewer #3: Yes

**********

5. Is the manuscript presented in an intelligible fashion and written in standard English?

Reviewer #3: Yes

**********

Reviewer #3: The revised manuscript demonstrates substantial improvement, and the authors have satisfactorily addressed the reviewers’ concerns. Therefore, I recommend it for publication as it is.

**********

what does this mean?). If published, this will include your full peer review and any attached files.

If you choose “no”, your identity will remain anonymous but your review may still be made public.

**Do you want your identity to be public for this peer review?**  For information about this choice, including consent withdrawal, please see our Privacy Policy

Reviewer #3: **Yes:** Youness El Bakri

**********

# Editor report: Acceptance letter

**DOI:** 10.1371/journal.pone.0352669.r010

**Authors:** Javed Iqbal

PONE-D-25-46615R4

PLOS One

Dear Dr. Alotaiq,

I'm pleased to inform you that your manuscript has been deemed suitable for publication in PLOS One. Congratulations! Your manuscript is now being handed over to our production team.

At this stage, our production department will prepare your paper for publication. This includes ensuring the following:

* All references, tables, and figures are properly cited

* All relevant supporting information is included in the manuscript submission,

* There are no issues that prevent the paper from being properly typeset

You will receive further instructions from the production team, including instructions on how to review your proof when it is ready. Please keep in mind that we are working through a large volume of accepted articles, so please give us a few days to review your paper and let you know the next and final steps.

Lastly, if your institution or institutions have a press office, please let them know about your upcoming paper now to help maximize its impact. If they'll be preparing press materials, please inform our press team within the next 48 hours. Your manuscript will remain under strict press embargo until 2 pm Eastern Time on the date of publication. For more information, please contact onepress@plos.org.

You will receive an invoice from PLOS for your publication fee after your manuscript has reached the completed accept phase. If you receive an email requesting payment before acceptance or for any other service, this may be a phishing scheme. Learn how to identify phishing emails and protect your accounts at https://explore.plos.org/phishing.

If we can help with anything else, please email us at customercare@plos.org.

Thank you for submitting your work to PLOS ONE and supporting open access.

Kind regards,

PLOS ONE Editorial Office Staff

on behalf of

Dr. Javed Iqbal

Academic Editor

PLOS One
